# Discovery of rare, diagnostic *Alu*Yb8/9 elements in diverse human populations

**DOI:** 10.1186/s13100-017-0093-0

**Published:** 2017-07-27

**Authors:** Julie Feusier, David J. Witherspoon, W. Scott Watkins, Clément Goubert, Thomas A. Sasani, Lynn B. Jorde

**Affiliations:** Department of Human Genetics, University of Utah School of Medicine, Salt Lake City, UT USA

**Keywords:** Retrotransposon, Mobilome, Polymorphism, Population genetics, Human ancestry, Ancestry informative markers

## Abstract

**Background:**

Polymorphic human *Alu* elements are excellent tools for assessing population structure, and new retrotransposition events can contribute to disease. Next-generation sequencing has greatly increased the potential to discover *Alu* elements in human populations, and various sequencing and bioinformatics methods have been designed to tackle the problem of detecting these highly repetitive elements. However, current techniques for *Alu* discovery may miss rare, polymorphic *Alu* elements. Combining multiple discovery approaches may provide a better profile of the polymorphic *Alu* mobilome. *Alu*Yb8/9 elements have been a focus of our recent studies as they are young subfamilies (~2.3 million years old) that contribute ~30% of recent polymorphic *Alu* retrotransposition events. Here, we update our ME-Scan methods for detecting *Alu* elements and apply these methods to discover new insertions in a large set of individuals with diverse ancestral backgrounds.

**Results:**

We identified 5,288 putative *Alu* insertion events, including several hundred novel *Alu*Yb8/9 elements from 213 individuals from 18 diverse human populations. Hundreds of these loci were specific to continental populations, and 23 non-reference population-specific loci were validated by PCR. We provide high-quality sequence information for 68 rare *Alu*Yb8/9 elements, of which 11 have hallmarks of an active source element. Our subfamily distribution of rare *Alu*Yb8/9 elements is consistent with previous datasets, and may be representative of rare loci. We also find that while ME-Scan and low-coverage, whole-genome sequencing (WGS) detect different *Alu* elements in 41 1000 Genomes individuals, the two methods yield similar population structure results.

**Conclusion:**

Current *in-silico* methods for *Alu* discovery may miss rare, polymorphic *Alu* elements. Therefore, using multiple techniques can provide a more accurate profile of *Alu* elements in individuals and populations. We improved our false-negative rate as an indicator of sample quality for future ME-Scan experiments. In conclusion, we demonstrate that ME-Scan is a good supplement for next-generation sequencing methods and is well-suited for population-level analyses.

**Electronic supplementary material:**

The online version of this article (doi:10.1186/s13100-017-0093-0) contains supplementary material, which is available to authorized users.

## Background

With >1.1 million copies, *Alu* elements are the most abundant and active retrotransposons in the human genome [1–3]. *Alu* elements are members of the SINE family of elements and utilize the LINE-1 endonuclease and reverse-transcriptase for retrotransposition [4]. This process inserts the *Alu* element, including its constitutive poly(A) tail and a target site duplication (TSD) sequence, into the genome [4, 5]. These hallmarks provide evidence of a retrotransposition event rather than a duplication or rearrangement.

While the vast majority of *Alu* elements in the human genome expanded during primate evolution and are no longer active, there are at least 42 retrotranspositionally active subfamilies today [6–10]. Furthermore, an active element with unique mutations has the potential to establish a new subfamily through retrotransposition in the genome [8, 10–12]. Recently retrotransposed *Alu* elements in some of these subfamilies are polymorphic for their presence or absence in the genome and are therefore useful for population and forensic analyses [7, 13–20]. *Alu* elements also contribute to the variation and regulation of the human genome [16, 18, 21–23], thus highlighting the importance of characterizing rare, ancestrally informative loci.

Detecting all polymorphic *Alu* elements in humans has been challenging for several reasons. First, the typical output from high-throughput sequencing are 100 bp paired end reads and do not completely cover the length of the 300-bp *Alu* element nor the flanking region necessary for proper mapping [13]. Second, *Alu* elements are commonly found within repetitive regions, which cause alignment errors and inaccurate mapping [18, 24, 25]. Third, the datasets analyzed thus far had insufficient coverage (e.g. 1000 Genomes Project has on average only ~7× per sample) to accurately assemble all *Alu* elements [13, 16, 18, 21]. Finally, different bioinformatics tools report different mobile element sets, and it appears that multiple tools are necessary to detect the whole mobilome [26, 27].

Mobile element scanning (ME-Scan), a method developed for mobile element discovery, attempts to addresses the mapping problem by allowing for high-coverage sequencing of the 5′ flank of the *Alu* breakpoint, the junction between the (unique) genomic sequence and the *Alu* element [28, 29]. ME-Scan can be modified to target specific mobile elements or subfamilies and can be applied in a wide variety of organisms [28–31]. In our study, ME-Scan targets the 7 bp insertion in *Alu*Yb8/9 elements, allowing subfamily-specific amplification and insertion detection using high-throughput sequencing protocols [28, 29, 32, 33]. The *Alu*Yb8/9 subfamilies are particularly interesting as they are young (~2.3 million years old) [34, 35] and active elements. Specifically, ~28% of the polymorphic *Alu* elements in a recent study [6] and ~33% of characterized disease-causing de novo *Alu* elements [36] are members of the Yb8 or Yb9 subfamilies. Here, we present an analysis of 213 individuals (including 43 from the 1000 Genomes (1KG) Project) from 18 diverse populations (Additional file 1: Table S1) using an updated protocol of ME-Scan. This refined examination allows us to characterize new rare *Alu*Yb8/9 elements, to analyze subfamilies, and to discover ancestrally informative markers. We also compare our detection of *Alu* elements to low-coverage, whole-genome sequenced datasets.

## Results

### Replicate and false positive analysis

We updated ME-Scan with standard Illumina primers to better facilitate library preparation and sequencing (Additional file 2: Supplemental Methods). Eleven independent replicates of an African Pygmy individual, AFP20, were sequenced via ME-Scan to assess run-to-run consistency and library quality. We performed locus-specific PCR to validate 22 non-reference insertions that were present in at least one AFP20 replicate but absent from the rest of the dataset (singletons) (Additional file 1: Tables S2, S3). Eight single-replicate insertions and two insertions with low read counts within SVA_D (SINE/VNTR/*Alu*) elements did not have an *Alu* element when detected by PCR. All remaining positions except one, which was located within a segmental duplication on chromosome 17, contained an *Alu* insertion. We also tested nine insertions found in AFP20 and in one other individual (doubletons), and all nine insertions were confirmed by PCR (Additional file 1: Tables S2, S3). We conclude that sequencing replicates may reduce false positives and improve the detection rate for singleton mobile element insertion events, the most difficult class of *Alu* elements to detect.

For assessment of sample quality, we filtered our previous set of presumably fixed *Alu* elements to 1601 elements that were not located within segmental duplications and highly likely to be fixed in the human genome (Additional file 1: Table S4). These loci should be easily detected by ME-Scan (Additional file 1: Table S4). We found that there is a linear inverse relationship between the false negative rate of these presumably fixed elements and the detection rate of the rare insertions in the AFP20 replicates (Additional file 3: Figure S1). Specifically, the replicates with less than a 10% false-negative rate had the highest (> = 75%) detection rate of the rare loci. Therefore, samples showing a false negative rate of more than 10% for these 1601 fixed loci are very likely to be of low quality.

Since most individuals in the study did not have replicates, it was necessary to establish a true positive threshold for all singletons and doubletons in the dataset. We PCR-validated 60 singleton (Additional file 3: Figure S2) and six doubleton loci (Additional file 1: Tables S2, S3). Building from our past studies [28, 29], the number of unique reads, instead of total read count, was the best indicator of a true-positive *Alu* insertion (Additional file 3: Figure S2). Based on these validation studies, a threshold of at least eight unique reads was required to call putative singleton and doubleton insertions. This resulted in a list of 5288 loci that were either previously established elements (Repbase *Alu*Y8/9) [37] or non-reference loci with at least eight unique reads in an individual.

We used principal components analysis (PCA) to examine the consistency between the population structure obtained with our updated protocol versus previously published protocols (Additional file 3: Figure S3) [28]. The Brahmin, YRI, and TSI samples were sequenced using different primers (*Alu*SPv2) than the rest of the samples (*Alu*SPv3) in the previously published dataset [28]. The second largest principal component (Additional file 3: Figure S3) separates the samples processed with different primers; however, there appears to be good consistency among the two datasets given this difference in primers. Therefore, we are confident in the updated protocol and our new criterion of sample quality.

### Identification of population-specific *Alu* elements

**Fig. 1:**
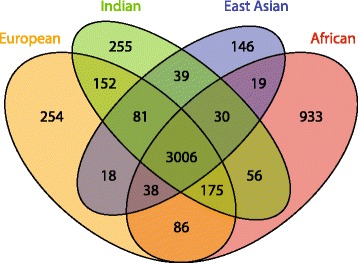
Venn diagram of *Alu* elements among 4 regional populations. Each individual was placed into one of four regional groups. Every putative locus per individual (5288 total loci) was added into the particular regional group

Ancestry-informative *Alu* elements can complement single nucleotide polymorphisms in detecting admixture or population structure [13, 15, 16]. In our cohort of 213 individuals, 30.03% of the 5288 loci (See Methods) are specific to one regional group (Fig. 1). We then sought to identify and characterize rare, population-specific *Alu* elements for ancestry studies. From the initial 5288 elements, loci found in our published analysis as well as the datasets we previously examined were removed (see Methods) [15, 28, 38–40]. To minimize false-positives due to mapping error, insertions that were within 50 bp of a reference *Alu* element or a simple repeat were also removed (BEDTools v2.19.1 Quinlan and Hall, 2010 [41]). This resulted in a list of 323 presumably population-specific loci: 117 from Africa, 103 from East Asia, 33 from India, and 70 from Europe (Additional file 1: Table S5, S6). We randomly selected 50 insertions and were able to design PCR primers and had sufficient DNA to test 30 candidate loci (Additional file 1: Tables S2, S3, S6). One primer set failed to amplify the predicted reference band, and the predicted reference element from the reference sample but no *Alu* fragment was amplified for 12 candidate loci. In total, 17/29 loci were true polymorphic *Alu* elements, including a novel *Alu* element on the Y chromosome (chrY:9,992,131 [hg19]) that was found in an East Asian individual. As expected, the validation rate (58%) for these very low frequency loci and singletons was lower than previous validation rates for common loci by ME-Scan [28, 29]. Because of our sample sizes, these insertions may not be truly population-specific but may be present at a higher copy frequency in one regional group than others.

The presence of a set of very rare *Alu* elements may be sufficient to classify an individual into a specific population. To identify diagnostic population-specific *Alu* elements, we genotyped six additional population-specific insertions with varying allele frequencies (0.006–0.087 in the specific population) (Additional file 1: Table S2, S3, S7) on a panel of 95 individuals (24 African, 24 European, 24 Indian, 23 East Asian) (Additional file 1: Table S8). Four of the loci were not detected in the population panel. An element (chr9:114,889,844 [hg19]) that was validated in two East Asian individuals from ME-Scan was detected in two additional East Asian individuals in the panel and absent in the other populations (Additional file 1: Tables S7, S8). This element may be more common than our analysis suggests (0.0303 with ME-Scan and 0.0357 with ME-Scan and Panel in East Asian individuals) because DNA was unavailable for three of the seven individuals detected for this locus by ME-Scan. Another element (chr9:114,940,676 [hg19]) was detected in nine copies in Africans via ME-Scan and was also present as a heterozygote in two African individuals and absent from the other populations in the panel (Additional file 1: Tables S7, S8). The minor copy frequency (0.0723) of this *Alu* element is statistically significantly different in the African population than the other populations (Wilson binomial 95% CI (0.0409–0.1250) for African, Wilson binomial 95% CI (0.0000–0.0332) for East Asian, the population with the lowest number of haploid genomes at this locus). These *Alu* elements are rare within one population group, may be absent or present at a very low copy number frequency in other populations, and add to a growing number of markers useful for ancestry studies.

### Discovery of *Alu* elements in exonic regions

*Alu* insertions inside exons are rare and often deleterious in humans [15, 32, 36], so we investigated non-reference exonic insertions in our dataset. We annotated candidate insertions by their presence or absence in noncoding and coding exonic regions [28]. We detected 17 loci within noncoding exonic regions and validated 3/3 polymorphic *Alu* elements within UTRs via PCR (Additional file 1: Tables S2, S3, S9). We also detected and designed primers for two candidate coding exonic loci (Additional file 1: Tables S10). Both primers amplified the expected reference band, and an *Alu* element was detected in one locus.

**Fig. 2:**
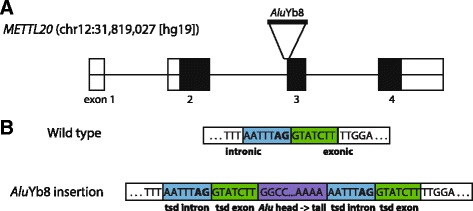
Diagram of an identified heterozygous *Alu* insertion in *METTL20*. **a**: Diagram of *Alu*Yb8 insertion in *METTL20*. Open boxes indicate untranslated regions, closed boxes indicate coding regions, and lines indicate intronic regions. **b**: Diagram of the WT and the *Alu*Yb8 insertion sequences in exon 3 of *METTL20*. The light blue indicates intronic TSD region, green indicates exonic TSD region, and purple is the *Alu* sequence. The insertion of the *Alu* element duplicated the AG splice acceptor site, indicated in bold font

We detected a heterozygous *Alu* insertion in exon 3 of *METTL20* (methyltransferase like 20) in the East Asian individual, 92–40-6 (Fig. 2a). *METTL20* was the first reported mitochondrial lysine methyltransferase characterized in animals and is thought to methylate non-histone proteins [42, 43]. Specifically, the *Alu*Yb8 element inserted near the start of the exon and duplicated the last seven nucleotides of the intron, including the AG splice acceptor site, as well as the first seven nucleotides of the exon as part of the TSD (Fig. 2b). This *Alu* element was also detected in the recent 1KG structural variation dataset [18] and appears to be present at very low frequencies (0.015 minor copy frequency in ME-Scan and 0.00097 minor copy frequency in 1KG) in the East Asian population. Further examination of the *METTL20* transcript will be required to determine if the *Alu* element is exonized through alternative slicing of the TSD AG splice acceptor site, thus potentially altering the function of this protein in some populations.

### Comparison of Yb8/9 elements detected by ME-Scan and WGS in individuals from the 1000 Genomes Project

**Fig. 3:**
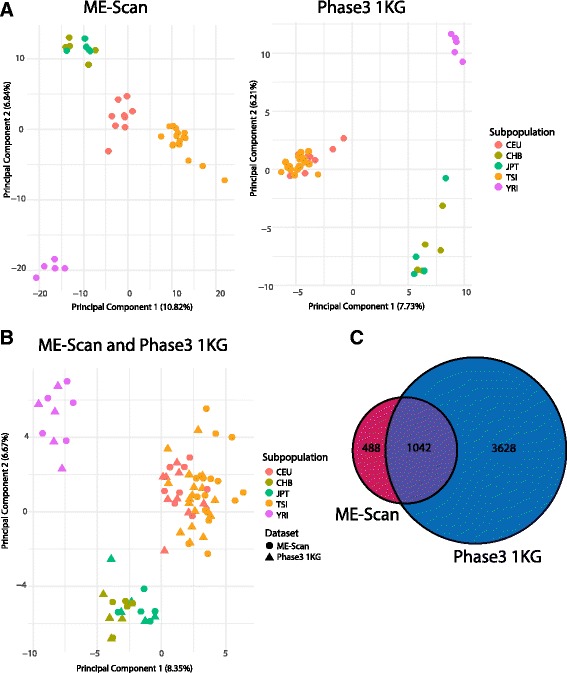
Comparison of *Alu* elements between ME-Scan and Phase3 datasets. **a**: On left, PCA of 41 1KG individuals (with less than 10% false negative rate) using 1266 polymorphic loci. Good *Alu* loci (Additional file 1: Table S4) and loci with presence/absence allele frequency of less than 5% or greater than 95% (all samples) were removed. On right, PCA of the same 41 IKG individuals with 2710 polymorphic *Alu* loci detected through the Phase3 WGS approach. Loci with presence/absence allele frequency for all individuals of less than 5% or greater than 95% were removed. **b**: PCA of 41 1KG individuals from both methods using the 191 shared loci from (**a)** *.* **c**: Venn Diagram of non-reference elements from ME-Scan and Phase3 in 1KG individuals. Phase3 dataset contains only polymorphic *Alu* elements, so ME-Scan loci were filtered to 1530 loci that were found in the 41 IKG individuals and absent from Repbase and build hg19. The Phase3 dataset was also filtered to 4670 *Alu* elements that were present in the 41 IKG individuals and absent from Repbase and build hg19. These two sets were then compared

Tens of thousands of polymorphic *Alu* elements have been discovered through the HapMap and 1KG consortiums [3, 18, 21, 44]. To assess consistency across platforms, we compared *Alu* elements found by ME-Scan to *Alu* (including non-Yb8/9) elements from the Phase3 1KG dataset in 41 1KG high-quality samples present in both datasets [18]. We performed PCA of these 41 1KG individuals using polymorphic *Alu* elements that were detected in either the ME-Scan or Phase3 datasets [18] (Fig. 3a). A PCA of 191 shared loci for both datasets reveals consistency between the two approaches (Fig. 3b).

Some *Alu* elements may have been missed in the low-coverage (~7×) WGS datasets [3, 18]. We examined the number of loci shared between the datasets to assess the concordance of the methods. The Phase3 dataset contains only polymorphic loci, so loci present in Repbase or reference build hg19 were removed from both datasets to attempt to address this bias. Each method found hundreds of unique loci in the 411KG individuals, as shown in Fig. 3c. Over 99.6% of the shared elements were either classified as *Alu*Yb elements or were not classified in the Phase3 dataset (Additional file 3: Figure S4). It is not surprising that there are thousands of unique loci in the Phase3 dataset compared to ME-Scan, given that the 1KG analysis did not target specific *Alu* subfamilies.

Next, we sought to determine whether ME-Scan detects novel *Alu* elements not detected by WGS in 1KG individuals. After comparison of multiple published datasets (see Methods) and filtering out false positive loci, 313 presumably novel *Alu* element insertions were identified in ME-Scan that had at least eight unique reads in at least one individual (Additional file 1: Tables S11, S12). Of these 313 presumably novel loci, 174 were detected in the 43 1KG individuals that were sequenced by ME-Scan (NA07346 and NA20515 were not in the comparison analyses) (Additional file 1: Table S12). Furthermore, a novel, validated population-specific *Alu* element (chr8:116,728,191 [hg19]) was found in TSI individual NA20518.

### Characterization of PCR-validated *Alu*Yb8/9 elements and identification of potential source elements

We performed Sanger sequencing and alignments of 68 validated rare *Alu*Yb8 (*N* = 58) and *Alu*Yb9 (*N* = 10) elements from the loci validated by PCR (Additional file 1: Tables S2, S13). Five *Alu* elements had a 5′ truncation of up to 20 bp, but the truncations did not impact subfamily identification (Additional file 1: Table S13). All elements had been correctly mapped to within 1 bp, after adjustment of 5′ modifications, of the predicted junction location (Additional file 1: Table S13). Fourteen and four of our loci were exact matches to the Yb8 and Yb9 consensus sequences, respectively (Additional file 1: Table S13). Nine of the 15 Yb8b1 elements were an exact match to Yb8b1 (a subfamily of Yb8) [6], and all three Yb11 elements (a subfamily of Yb9) were an exact match to Yb11 [35]. Because we targeted *Alu* elements with the 7 bp insertion that is diagnostic of many *Alu*Yb subfamilies, it was not surprising that eight of the elements belonged to other Yb subfamilies. The elements diverged from their respective consensus subfamily by an average of 0.431% (+/− 0.635 s.d.), and 45.5% of the elements were full-length and an exact match to the consensus sequence based on BLAST+ analysis (Additional File 1: Table S12, see distribution in Additional file 3: Figure S5) [45]. The *Alu* and flanking sequence for each locus is presented in Additional file 4.

**Fig. 4:**
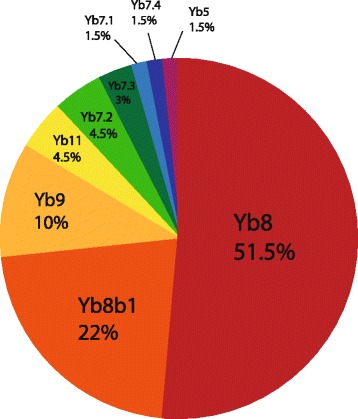
Subfamily distribution of 68 Sanger-sequenced *Alu*Yb elements

We examined the distribution of our Sanger-sequenced elements among the *Alu*Yb subfamilies (Fig. 4) and compared this to a previous *Alu*Yb subfamily distribution analysis [6]. Notably, we detected more elements that belong to the recently characterized *Alu*Yb8 subfamily, *Alu*Yb8b1, than in [6]. However, the proportion of Yb8b1 elements between the datasets was not significantly different (Fisher exact test, *P* > 0.186). Furthermore, the difference in the proportion of *Alu*Yb8 elements (the only other subfamily that possibly differed) between the datasets was also not statistically significant (Fisher exact test, *P* > 0.318). Therefore, we conclude that this distribution is similar to the Yb8/9 subfamily distribution in [6].

Active *Alu* elements have the potential to retrotranspose in the genome; these “source” elements have at least four characteristic hallmarks: intact box A and B internal RNA Polymerase III (pol III) promoters [10, 11, 46], intact SRP9/14 sites [11], a poly(A) tail at least 20 bases long (preferably uninterrupted As) [12], and a pol III termination sequence, TTTT, within 15 bp of the TSD downstream of the poly(A) tail [46]. Sixty-two of the 68 Sanger-sequenced *Alu* elements had enough sequence information via Sanger sequencing for analysis of these hallmarks. Sequences typically terminate within the poly(A) tail during Sanger sequencing; however, we estimated an approximate length for each poly(A) tail (Additional file 1: Table S13, Additional file 4). Only one element had an A-tail shorter than 20 bp, and another element had evidence of substitutions within the poly(A) tail. Overall, we detected 11 elements across five *Alu*Yb subfamilies that contained the hallmarks of potential source elements (Additional file 1: Table S13). Potential source elements are difficult to accurately detect, and other factors like the 5′ flanking sequence are important for pol III transcription [47, 48]; however, these 11 elements are the most likely candidates in this dataset.

## Discussion

In this analysis, we present and utilize an updated version of a recently developed mobile element scanning technique, ME-Scan [28, 29], to examine *Alu*Yb8/9 elements in world-wide human populations. Our updated method is consistent with the previous ME-Scan protocol (Additional file 3: Figure S3) [28] and standardizes the entire ME-scan protocol for use on the Illumina HiSeq2000 without instrument adjustments (Additional file 2). We also present sequence information for 68 rare *Alu*Yb8/9 elements (Additional file 4), including 23 presumably population-specific loci and 11 elements with hallmarks of active source elements. Furthermore, ME-Scan is able to detect hundreds of *Alu* insertions previously not found by non-targeted high-throughput sequencing methods, thus demonstrating a clear utility for multiple approaches to fully characterize the mobilome.

Discovery of rare, polymorphic *Alu* elements can be useful for distinguishing human ancestral identity. One key limiting factor, particularly with population-specific loci, is the number of new individuals being studied. With 213 individuals in this dataset, 74% and 13% of the population-specific loci were singletons and doubletons, respectively. This indicates that there may be hundreds, and potentially thousands, of unidentified *Alu* elements present at low minor allele frequencies in the human population, and potentially private mutations through de novo retrotransposition (the current expected de novo mutation rate is ~1:20 births [49]). Additionally, all of the Sanger-sequenced *Alu*Yb8/9 elements were present as heterozygotes in ME-Scan individuals, with the exception of *Alu*Yb8 at position chr9:114,889,844 [hg19], which was found as a homozygote in one individual and a heterozygote in seven individuals (Additional file 1: Table S9, S10). The preliminary findings from testing six rare, population-specific loci on a PCR panel of 95 individuals revealed that these loci may be diagnostic of specific populations, as they were present at a low allele frequency in one population (0.0049–0.0724) and absent from the rest. This finding also highlights the sensitivity of our method for detecting rare *Alu* insertions. Further examination of large cohorts will reveal additional diagnostic loci, as the majority of high-frequency *Alu* elements in the human genome have already been identified.

*Alu* discovery is challenging due to mapping/alignment errors and low sequencing depth of repetitive regions [26, 27]. The majority of the tested loci identified in these analyses were located in repetitive regions, and the false-positive loci may be due to alignment or PCR artifacts. Additionally, six of the 12 false-positive population-specific loci were singletons in individuals with >10% false negative rates. This helps to validate our criterion that >10% false negative rate indicates a poor-quality sample. Another two of the 12 false-positive loci were also detected in different individuals in the Phase3 1KG dataset [18]. This could be due to sample identity error, technical, mapping, or PCR artifacts, but it underscores the fact that PCR is still an important validation component of next-generation sequencing approaches.

*Alu* subfamily classification is an active field, and at least six new subfamilies have been classified in recent years [6, 35]. One goal of this project was to characterize the subfamily distribution of rare *Alu* elements and potentially identify very young subfamilies. Notably, 15 of our 68 elements belong to the *Alu*Yb8b1 subfamily, adding support to the classification of this new subfamily [6]. Another interesting discovery was that our subfamily distribution of polymorphic Yb8/9 elements recapitulated the distribution from a previous study [6]. Thus, we conclude that ME-Scan does not appear to be biased within subfamilies with the 7 bp insertion.

In search of new subfamilies, we identified preliminary evidence of a novel subfamily in the *Alu*Yb8 lineage. We identified four loci that differed from the Yb8 reference sequence with C to T substitutions at CpG sites on positions 207, 213, and 258. A BLAT search of this sequence in both [hg19] and human genome build [38] revealed one exact match, and alignments to a previously published dataset [6] revealed two additional loci (*Alu* 161 and *Alu* 356 from [6]). The sequence’s location in the [hg19] reference genome (chr9:98,266,017 [hg19]) has the hallmark of an active source element, indicating that this subfamily may be currently active. However, CpG sites mutate at a rate six to ten times faster than non-CpG sites, and these mutations may have occurred after retrotransposition [9, 50, 51]. Classification of active subfamilies through de novo or somatic retrotransposition events rather than from sequence information would help to answer this question, as this would eliminate mutations that occur after retrotransposition. Further evidence will be needed to determine whether these three CpG mutations are diagnostic of a novel subfamily, *Alu*Yb8c3, or a collection of independent, random events.

For population genomics analyses, we demonstrate that PCA results based on ME-Scan compare almost perfectly to those of WGS approaches (Fig. 3a, b) [16, 18]. Platform differences did not seem to be involved in the first two principal components of the PCA of the 1KG in the ME-Scan and Phase3 datasets (Fig. 3b). However, TSI does not cluster with CEU in the *Alu*Yb8/9 loci from ME-Scan, whereas TSI and CEU cluster together using loci from different *Alu* subfamilies in the Phase3 dataset (Fig. 3a). This is likely due to a library bias in ME-Scan, as the TSI were sequenced in a different library than the rest of the 1KG individuals. We also found that there are hundreds of unique loci in the 41 1KG individuals in either dataset (Fig. 3c). These results demonstrate that complementary methods, such as WGS and ME-Scan, provide a more complete genomic assessment of the *Alu* mobilome than either method alone.

## Conclusions

Here we demonstrate that ME-Scan detection is consistent with WGS approaches and is an independent complementary method for *Alu*Yb8/9 discovery. The updated protocol and threshold criteria allow for future studies to be performed with relative ease. Even as the cost of WGS continues to decrease, we conclude that ME-Scan provides alternate options in the field of transposable element population genomics and is scalable from pilot experiments to much larger projects involving the analysis of polymorphisms in hundreds of individuals.

## Methods

### DNA samples and ME-Scan protocol

The ME-Scan protocol was standardized to the Illumina HiSeq 2000 platform. A detailed report of the protocol including primers is provided in the Additional file 4: Supplemental Methods. Data were mapped to hg19 using bwa align (bwa version 0.7.9a) [52] and uploaded to SQL developer for analysis. Read set processing was the same as described in [28].

Two hundred thirty-three samples (213 unique individuals) were sequenced using the ME-Scan protocol and Illumina sequencing. These individuals were sampled from 21 groups, including 18 geographical ancestry groups: 6 Nande, 5 YRI (Yoruba in Ibadan, Nigeria), 16 Hema, and 24 Pygmy from sub-Saharan Africa; 22 Brahmin, 2 Irula, 2 Kapu, 2 Khonda Dora, 20 Madiga, 26 Mala, 2 Relli and 2 Yadava from south India; 18 TSI (Toscani in Italy), 10 CEU (CEPH samples from Utah) and 23 Europeans from west Europe; 5 CHB (Han Chinese in Beijing, China), 5 JPT (Japanese in Tokyo, Japan), 8 Japanese, 4 Vietnamese, an individual from Taiwan, and 10 individuals of mixed Asian ancestry from east Asia. DNA from 43 individuals (TSI, CEU, CHB, JPT, and YRI) was obtained from transformed lymphoblast cells lines from the HapMap Project [53]. DNA was obtained from whole blood for the remaining individuals (including the PCR panel), who have been described previously [54–56]. DNA for the TSI 1000 Genomes individuals and non-1000 Genomes individuals were available for PCR validation. Most of the indexed individuals were combined into 9 pooled libraries of ~25 individuals per library, designated AFHAFN, ASIAN, BRA, CAUC, HapMap, MADIGA, MALA, PYG, and TSI. Twenty-two indexed samples were combined into 5 pooled libraries that contained samples that were not part of this study, with ~53 individuals per library. These libraries were arbitrarily named Library 10–14 for this study.

### PCR validation and oligonucleotide primer design

Each locus was viewed on the hg19 build on the UCSC genome browser [57]. The DNA sequence was obtained with 500 bp of flanking sequence upstream and downstream of the potential breakpoint and was entered into primer-Blast and verified by in silico PCR from the UCSC genome browser [58]. In cases where primer-Blast was unable to create a primer set, the sequence was entered into Primer3 and the primer set was verified in primer-Blast [58, 59].

PCR amplifications of ~25 ng of template DNA were performed in 25 μl reactions according to the Phusion HotStart Flex DNA Polymerase protocol (using 5× GC buffer), with the exception that the quantity of 10 μM primers was reduced to 1 μl each. The thermocycler conditions were: initial denaturation at 98C for 20s, 34 cycles of denaturation at 98C for 20s, optimal annealing temperature (58–62) at 30s, extension at 72C for 30s, and a final extension at 72C for 7 min. Every primer set reaction was performed on the individual(s) with the candidate *Alu* element, a positive control (an individual not expected to contain the *Alu* element) and H_2_O. PCR amplicons of 24 μl were run on a 2% gel containing 0.12 mg/ml ethidium bromide for 60 min at 160 V. Gels were imaged using a Fotodyne Analyst Investigator Eclipse machine.

### Sanger sequencing

PCR amplicons of 20 μl per loci were run on a 2% agarose gel. The band that was shifted ~300 bp above the wildtype band was cut out and purified for sequencing using the Qiaquick gel extraction kit (Qiagen). When the candidate *Alu* element was present in multiple individuals, the DNA was pooled prior to purification. A total of 9.5 μl of purified DNA and 0.5 μl of 10 μM primer were used for Sanger sequencing. Each *Alu* element was also sequenced using an internal *Alu*Yb primer (ACGGAGTCTCGCTCTGTCG) that starts near the poly(A) tail and continues through the head of the *Alu* into the flanking region for double coverage of most of the *Alu* element. All Sanger sequencing reads were analyzed using Sequencher [60].

### Matching ME-Scan reads to reference genomes and published datasets

We matched the ME-Scan sequenced loci to the RepeatMasker-annotated hg19 reference genome [38], as in [28]. The positions were not corrected for possible 5′ truncations. Therefore, we added a 30 bp buffer upstream of the breakpoint was on the correct strand. We also compared the loci to dbRIP and two datasets for PCR validation [15, 21, 40]. The exonic regions were annotated as in [28]. We did not remove previously published ME-Scan-identified loci, as those had not been validated, with the exception of discovering population-specific loci. A putative list was made of loci that matched “*Alu*Yb8” or “*Alu*Yb9” by RepeatMasker or had at least eight unique reads in an individual (Additional file 1: Table S5).

After PCR validation, we further compared our results with recently published datasets to identify unpublished *Alu*Yb8/9 elements (Additional file 1: Tables S11, S12) [6, 13, 18, 28, 35, 61]. We extended the reference range to within 30 bp on either side of the ME-Scan breakpoint position for comparison with non-Repbase datasets. Additionally, we used the liftOver tool [57] in the UCSC genome browser to compare the build [hg38] *Alu* elements with these loci. Matches for all datasets are reported in Additional file 1: Table S10 and the novel loci are reported in Additional file 1: Table S11.

1KG *Alu* elements were downloaded from the Phase3 data release of the 1000 Genomes Project [3, 18, 62] (ftp://ftp.1000genomes.ebi.ac.uk/vol1/withdrawn/phase3/integrated_sv_map/ALL.wgs.integrated_sv_map_v2.20130502.svs.genotypes.vcf.gz).

## Additional files

Additional file 1: Tables S1-S12. This file contains supplementary Tables S1-S13 as well as a table of contents with table names (XLSX 1249 kb)

Additional file 2: Supplementary Methods. This file contains supplementary methods that contain the improved ME-Scan protocol (DOCX 29 kb)

Additional file 3: This file contains Figures S1-S5 and figure legends. (PDF 4383 kb)

Additional file 4: FASTA sequences of 68 *Alu* elements. This file contains high-quality sequence from Sanger sequencing of 68 *Alu* elements. The nucleotides are color-coded for *Alu*, TSD, A and B boxes, SRP9/14 sites, and pol III termination signals (DOCX 34 kb)

## Abbreviations

1KG: 1000 Genomes project
pol III: RNA Polymerase III
SINE: Short interspersed nuclear element
TSD: Target site duplication

## References

[1] Hasler J, Strub K. Alu elements as regulators of gene expression. Nucleic Acids Res. 2006;34:5491–7. 10.1093/nar/gkl706 PMC1636486 17020921

[2] The International Human Genome Mapping Consortium. A physical map of the human genome. Nature 2001;409:934–41. 10.1038/35057157 11237014

[3] The 1000 Genomes Project Consortium. A global reference for human genetic variation. Nature. 2015;526:68–74. 10.1038/nature15393 PMC4750478 26432245

[4] Dewannieux M, Esnault C, Heidmann T. LINE-mediated retrotransposition of marked Alu sequences. Nat Genet. 2003;35:41–8. 10.1038/ng1223 12897783

[5] Christensen SM, Eickbush TH. R2 target-primed reverse transcription: ordered cleavage and polymerization steps by protein subunits asymmetrically bound to the target DNA. Mol Cell Biol. 2005;25:6617–28. 10.1128/MCB.25.15.6617-6628.2005 PMC1190342 16024797

[6] Konkel MK, Walker JA, Hotard AB, Ranck MC, Fontenot CC, Storer J, et al. Sequence Analysis and Characterization of Active Human Alu Subfamilies Based on the 1000 Genomes Pilot Project. Genome Biol Evol. 2015;7:2608–22. 10.1093/gbe/evv167 PMC4607524 26319576

[7] Batzer MA, Deininger PL. Alu Repeats and Human Genomic Diversity. Nat Rev Genet. 2002;3:370–9. 10.1038/nrg798 11988762

[8] Han K, Xing J, Wang H, Hedges DJ, Garber RK, Cordaux R, et al. Under the genomic radar: The Stealth model of Alu amplification. Genome Res. 2005;15:655–64. 10.1101/gr.3492605 PMC1088293 15867427

[9] Xing J, Hedges DJ, Han K, Wang H, Cordaux R, Batzer M A Alu element mutation spectra: molecular clocks and the effect of DNA methylation. J Mol Biol. 2004;344:675–82. 10.1016/j.jmb.2004.09.058 15533437

[10] Mills RE, Bennett EA, Iskow RC, Devine SE. Which transposable elements are active in the human genome? Trends Genet. 2007;23:183–91. 10.1016/j.tig.2007.02.006 17331616

[11] Bennett EA, Keller H, Mills RE, Schmidt S, Moran JV, Weichenrieder O, et al. Active Alu retrotransposons in the human genome. Genome Res. 2008;18:1875–83. 10.1101/gr.081737.108 PMC2593586 18836035

[12] Dewannieux M, Heidmann T. Role of poly(A) tail length in Alu retrotransposition. Genomics. 2005;86:378–81. 10.1016/j.ygeno.2005.05.009 15993034

[13] Wildschutte JH, Baron AA, Diroff NM, Kidd JM. Discovery and characterization of Alu repeat sequences via precise local read assembly. Nucleic Acids Res. 2015;43:10292–307. 10.1093/nar/gkv1089 PMC4666360 26503250

[14] Tajnik M, Vigilante A, Braun S, Hänel H, Luscombe NM, Ule J, et al. Intergenic *Alu* exonisation facilitates the evolution of tissue-specific transcript ends. Nucleic Acids Res. 2015;43:gkv956. 10.1093/nar/gkv956 PMC4666398 26400176

[15] Hormozdiari F, Alkan C, Ventura M, Hajirasouliha I, Malig M, Hach F, et al. Alu repeat discovery and characterization within human genomes. Genome Res. 2011:840–9. 10.1101/gr.115956.110 PMC3106317 21131385

[16] Rishishwar L, Tellez Villa CE, Jordan IK. Transposable element polymorphisms recapitulate human evolution. Mobile DNA. 2015;6:21. 10.1186/s13100-015-0052-6 PMC4647816 26579215

[17] Salem AH, Kilroy GE, Watkins WS, Jorde LB, Batzer MA. Recently integrated Alu elements and human genomic diversity. Mol Biol Evol. 2003;20:1349–61. 10.1093/molbev/msg150 12777511

[18] Sudmant PH, Rausch T, Gardner EJ, Handsaker RE, Abyzov A, Huddleston J, et al. An integrated map of structural variation in 2,504 human genomes. Nature. 2015;526:75–81. 10.1038/nature15394 PMC4617611 26432246

[19] Gu Z, Jin K, Crabbe MJC, Zhang Y, Liu X, Huang Y, et al. Enrichment analysis of Alu elements with different spatial chromatin proximity in the human genome. Protein Cell. Higher Education Press. 2016;7:250–66. 10.1007/s13238-015-0240-7 PMC4818845 26861146

[20] Wang L, Rishishwar L, Mariño-Ramírez L, Jordan IK. Human population-specific gene expression and transcriptional network modification with polymorphic transposable elements. Nucleic Acids Res. 2017;45:2318–28. 10.1093/nar/gkw1286 PMC5389732 27998931

[21] Stewart C, Kural D, Strömberg MP, Walker JA, Konkel MK, Stütz AM, et al. A comprehensive map of mobile element insertion polymorphisms in humans. PLoS Genet. 2011;7:e1002236. 10.1371/journal.pgen.1002236 PMC3158055 21876680

[22] Witherspoon DJ, Watkins WS, Zhang Y, Xing J, Tolpinrud WL, Hedges DJ, et al. Alu repeats increase local recombination rates. BMC Genomics. 2009;10:530. 10.1186/1471-2164-10-530 PMC2785838 19917129

[23] Xing J, Zhang Y, Han K, Xing J, Zhang Y, Han K, et al. Mobile elements create structural variation: Analysis of a complete human genome. Genome Res. 2009:1516–26. 10.1101/gr.091827.109 PMC2752133 19439515

[24] Thung DT, de Ligt J, Vissers LEM, Steehouwer M, Kroon M, de Vries P, et al. Mobster: accurate detection of mobile element insertions in next generation sequencing data. Genome Biol. 2014;15:488. 10.1186/s13059-014-0488-x PMC4228151 25348035

[25] Wu J, Lee W-P, Ward A, Walker JA, Konkel MK, Batzer MA, et al. Tangram: a comprehensive toolbox for mobile element insertion detection. BMC Genomics. 2014;15:795. 10.1186/1471-2164-15-795 PMC4180832 25228379

[26] Ewing AD. Transposable element detection from whole genome sequence data. Mobile DNA. 2015;6:24. 10.1186/s13100-015-0055-3 PMC4696183 26719777

[27] Rishishwar L, Mariño-Ramírez L, Jordan IK. Benchmarking computational tools for polymorphic transposable element detection. Brief Bioinform. 2016;bbw072:1–11. 10.1093/bib/bbw072 PMC5808724 27524380

[28] Witherspoon DJ, Zhang YH, Xing JC, Watkins WS, Ha H, Batzer MA, et al. Mobile element scanning (ME-Scan) identifies thousands of novel Alu insertions in diverse human populations. Genome Res. 2013;23:1170–81. 10.1101/gr.148973.112 PMC3698510 23599355

[29] Witherspoon DJ, Xing J, Zhang Y, Watkins WS, Batzer MA, Jorde LB. Mobile element scanning (ME-Scan) by targeted high-throughput sequencing. BMC Genomics. 2010;11:410. 10.1186/1471-2164-11-410 PMC2996938 20591181

[30] Platt RN, Zhang Y, Witherspoon DJ, Xing J, Suh A, Keith MS, et al. Targeted Capture of Phylogenetically Informative Ves SINE Insertions in Genus Myotis. Genome Biol Evol. 2015;7:1664–75. 10.1093/gbe/evv099 PMC4494050 26014613

[31] Ha H, Loh JW, Xing J. Identification of polymorphic SVA retrotransposons using a mobile element scanning method for SVA (ME-Scan-SVA). Mobile DNA. 2016;7:15. 10.1186/s13100-016-0072-x PMC4967303 27478512

[32] Xing J, Witherspoon DJ, Jorde LB. Mobile element biology: new possibilities with high-throughput sequencing. Trends Genet Elsevier Ltd. 2013;29:280–9. 10.1016/j.tig.2012.12.002 PMC3938198 23312846

[33] Ha H, Wang N, Xing J. Library Construction for High-Throughput Mobile Element Identification and Genotyping. Methods Mol Biol. Totowa, NJ: Humana Press; 2015. p. 1–15. 10.1007/7651_2015_265 26025622

[34] Carter AB, Salem A, Hedges DJ, Keegan CN, Kimball B, Walker JA, et al. Genome-wide analysis of the human Yb-lineage. Hum Genomics. 2004;1:167–78. 10.1186/1479-7364-1-3-167 PMC3525081 15588477

[35] Ahmed M, Li W, Liang P. Identification of three new Alu Yb subfamilies by source tracking of recently integrated Alu Yb elements. Mobile DNA. 2013;4:25. 10.1186/1759-8753-4-25 PMC3831846 24216009

[36] Hancks DC, Kazazian HH. Roles for retrotransposon insertions in human disease. Mobile DNA. 2016;7:9. 10.1186/s13100-016-0065-9 PMC4859970 27158268

[37] Jurka J, Kapitonov VV, Pavlicek A, Klonowski P, Kohany O, Walichiewicz J. Repbase Update, a database of eukaryotic repetitive elements. Cytogenet Genome Res. 2005;110:462–7. 10.1159/000084979 16093699

[38] Smit A, Hubley R, Green P. RepeatMasker Open-3.0. 2010.

[39] Stewart C, Kural D, Strömberg MP, Walker JA, Konkel MK, Stütz AM, et al. A comprehensive map of mobile element insertion polymorphisms in humans. PLoS Genet. 2011;7 10.1371/journal.pgen.1002236 PMC3158055 21876680

[40] Wang J, Song L, Grover D, Azrak S, Batzer MA, Liang P. dbRIP: A Highly Integrated Database of Retrotransposon Insertion Polymorphisms in Humans. Hum Mutat. 2006;27:323–9. 10.1002/humu.20307 PMC1855216 16511833

[41] Quinlan AR, Hall IM. BEDTools: a flexible suite of utilities for comparing genomic features. Bioinformatics. 2010;26:841–2. 10.1093/bioinformatics/btq033 PMC2832824 20110278

[42] Cloutier P, Lavallée-Adam M, Faubert D, Blanchette M, Coulombe B. A Newly Uncovered Group of Distantly Related Lysine Methyltransferases Preferentially Interact with Molecular Chaperones to Regulate Their Activity. PLoS Genet. 2013;9:e1003210. 10.1371/journal.pgen.1003210 PMC3547847 23349634

[43] Małecki J, Ho AYY, Moen A, Dahl H-A, Falnes PØ. Human METTL20 is a mitochondrial lysine methyltransferase that targets the β subunit of electron transfer flavoprotein (ETFβ) and modulates its activity. J Biol Chem. 2015;290:423–34. 10.1074/jbc.M114.614115 PMC4281744 25416781

[44] The 1000 Genomes Project Consortium. A map of human genome variation from population-scale sequencing. Nature. 2010;467:1061–73. 10.1038/nature09534 PMC3042601 20981092

[45] Camacho C, Coulouris G, Avagyan V, Ma N, Papadopoulos J, Bealer K, et al. BLAST+: architecture and applications. BMC Bioinformatics. 2009;10:421. 10.1186/1471-2105-10-421 PMC2803857 20003500

[46] Comeaux MS, Roy-Engel AM, Hedges DJ, Deininger PL. Diverse cis factors controlling Alu retrotransposition: what causes Alu elements to die? Genome Res. 2009;19:545–55. 10.1101/gr.089789.108 PMC2665774 19273617

[47] Parekh RB, Dwek R, Sutton B. Upstream sequences modulate the internal promoter of the human 7SL RNA gene. Nature. 1985;318:452–7. 10.1038/318371a0 2415825

[48] Chesnokov I, Schmid CW. Flanking sequences of an Alu source stimulate transcription in vitro by interacting with sequence-specific transcription factors. J Mol Evol. 1996;42:30–6. 10.1007/BF00163208 8576961

[49] Cordaux R, Hedges DJ, Herke SW, Batzer MA. Estimating the retrotransposition rate of human Alu elements. Gene. 2006;373:134–7. 10.1016/j.gene.2006.01.019 16522357

[50] Batzer MA, Kilroy GE, Richard PE, Shaikh TH, Desselle TD, Hoppens CL, et al. Structure and variability of recently inserted Alu family members.[erratum appears in Nucleic Acids Res 1991 Feb 11;19(3):698–9]. Nucleic Acids Res. 1990;18:6793–8. 10.1093/nar/18.23.6793 PMC332733 2175877

[51] Labuda D, Striker G. Sequence conservation in Alu evolution. Nucleic Acids Res. 1989;17:2477–91. 10.1093/nar/17.7.2477 PMC317637 2541408

[52] Li H, Durbin R. Fast and accurate short read alignment with Burrows-Wheeler transform. Bioinformatics. 2009;25:1754–60. 10.1093/bioinformatics/btp324 PMC2705234 19451168

[53] The International HapMap 3 Consortium, Principal investigators, Altshuler DM, Gibbs RA, Peltonen L, Project coordination leaders, et al. Integrating common and rare genetic variation in diverse human populations. Nature. 2010;467:52–8. 10.1038/nature09298 PMC3173859 20811451

[54] Bamshad MJ, Watkins WS, Dixon ME, Jorde LB, Rao BB, Naidu JM, et al. Female gene flow stratifies Hindu castes. Nature. 1998;395:651. 10.1038/27103 9790184

[55] Jorde LB, Bamshad MJ, Watkins WS, Zenger R, Fraley AE, Krakowiak PA, et al. Origins and affinities of modern humans: a comparison of mitochondrial and nuclear genetic data. Am J Hum Genet. 1995;57:523–38. 10.1002/ajmg.1320570340 PMC1801280 7668280

[56] Watkins WS, Bamshad M, Dixon ME, Bhaskara Rao B, Naidu JM, Reddy PG, et al. Multiple Origins of the mtDNA 9-bp Deletion in Populations of South India. Am J Phys Anthropol. 1999;109(147–15):147–58. 10.1002/(SICI)1096-8644(199906)109:2<147::AID-AJPA1>3.0.CO;2-C 10378454

[57] Kent WJ, Sugnet CW, Furey TS, Roskin KM, Pringle TH, Zahler AM, et al. The Human Genome Browser at UCSC The Human Genome Browser at UCSC. Genome Res. 2002;12:996–1006. 10.1101/gr.229102 PMC186604 12045153

[58] Ye J, Coulouris G, Zaretskaya I, Cutcutache I, Rozen S, Madden TL. Primer-BLAST: A tool to design target-specific primers for polymerase chain reaction. BMC Bioinformatics. 2012;13:134. 10.1186/1471-2105-13-134 PMC3412702 22708584

[59] Untergasser A, Cutcutache I, Koressaar T, Ye J, Faircloth BC, Remm M, et al. Primer3-new capabilities and interfaces. Nucleic Acids Res. 2012;40:1–12. 10.1093/nar/gks596 PMC3424584 22730293

[60] Corporation GC. Sequencher. Ann Arbor: Gene Codes Corporation; 2015.

[61] David M, Mustafa H, Brudno M. Detecting Alu insertions from high-throughput sequencing data. Nucleic Acids Res. 2013;41:1–13. 10.1093/nar/gkt612 PMC3783187 23921633

[62] Durbin RM, Altshuler DL, Durbin RM, Abecasis GR, Bentley DR, Chakravarti A, et al. A map of human genome variation from population-scale sequencing. Nature. 2010;467:1061–73. 10.1038/nature09534 PMC3042601 20981092

