# Supplementary material for: Discovery of rare, diagnostic AluYb8/9 elements in diverse human populations
Source: Mob DNA. 2017 Jul 27;8:9. doi: 10.1186/s13100-017-0093-0 (PMC5531096; doi:10.1186/s13100-017-0093-0)
Supplement: Supplementary file 2 — This file contains supplementary methods that contain the improved ME-Scan protocol (DOCX 29 kb) [file 13100_2017_93_MOESM2_ESM.docx]

Supplementary Methods

*Sequencing adaptor and primer design for ME-Scan*

To facilitate rapid library preparation and sequencing, the ME-scan protocol was optimized and standardized to the Illumina HiSeq 2000 platform using commercially available reagents and modified Illumina sequencing adaptors and primers. The initial library construction was performed using a modified Illumina P7 adaptor. This adaptor has a custom 6 bp index1 barcode sequence that replaces the standard Illumina index1 and a second 6 bp index2 barcode sequence at the 3’ end. Each barcoded P7 primer and a complementary 25 bp primer were annealed by heating to 65^o^C and slowly cooling to ambient temperature over 30 minutes to create unique P7 adaptors. The *Alu*Yb8_short primer selectively amplifies *Alu*Yb8 and *Alu*Yb9 retrotransposons from the pooled libraries. The 3’-end of the *Alu*Yb8_short primer anneals to a 7 bp insertion unique to the Yb8 and Yb9 classes of *Alu* retrotransposons, and a biotinylated 5’-end enables the recovery of *Alu*-containing fragments after pooled library amplification. To allow for standard Illumina paired-end sequencing, the Illumina P5 primer was modified to contain a semi-random variable-length sequence immediately 3’ to the annealing site of the P5 sequencing primer. These random bases are the first to be sequenced with the P5 sequencing primer and prevent clustering errors that would otherwise result from the nearly identical bases in all *Alu*Yb8/9 elements. A full listing of all the primers is provided in supplementary methods table 1.

*Library construction*

Genomic DNA was prepared from venous blood samples using standard extraction protocols. Prior to library preparation, DNA samples were checked for degradation by electrophoresis on a 0.6% agarose gel. For each sample, three micrograms of high molecular weight DNA was transferred to a 6mm crimp-cap microtube and sheared to approximately 800-1000 bp fragments in a Covaris S220 focused-ultrasonicator (duty cycle: 5%; intensity: 3; cycles/burst: 200; time: 15 seconds).

Following shearing, fragment ends were repaired using a mixture of T4 DNA polymerase, T4 polynucleotide kinase, and Klenow fragment [fragmented sample DNA (30 ng/ul), T4 DNA polymerase (0.09 U/ul), T4 polynucleotide kinase (0.3 U/ul), Klenow fragment (0.03 U/ul), 0.4 mM each dNTPs, 50 mM Tris-HCl, 10 mM MgCl_2_, 1 mM ATP, 10 mM DTT, pH 7.5] at 30^o^C for 30 minutes in an ABI 9800 thermal cycler in a total reaction volume of 100 ul. Samples were processed in batches of twenty-four or forty-eight plus controls. All reactions were held on ice in an *Alu*minum block during preparation and then transferred directly to a pre-heated thermal cycler. End-repaired samples were purified away from the reaction mix using 1.8X volumes of AMPure XP magnetic beads. Beads were separated from the supernatant on an Ambion 96-well magnetic stand, washed 2X with 80% ethanol, and eluted in 10 mM Tris-HCl, pH 7.5.

To allow efficient ligation of the modified P7 adaptor, all recovered blunt-end genomic fragments were dA tailed with Klenow fragment (exo -) [0.18 U/ul Klenow fragment (exo -), 0.2 mM ATP, 50 mM NaCl, 10 mM Tris-HCl, 10 mM MgCl_2_, 1mM DTT, pH 7.9] at 37^o^C for 30 minutes in a total volume of 50 ul. Samples were purified with magnetic beads and eluted in 15 ul of 10 mM Tris-HCl. Barcoded P7 adaptors were ligated to all recovered A-tailed genomic fragments [NEB Quick ligase (24 U/ul), 0.2 uM barcoded adaptor primer, 66 mM Tris-HCl, 10 mM MgCl_2_, 1 mM DTT, 1 mM ATP, 6% PEG 6000, pH 7.6] at 20^o^C for 15 minutes in a total reaction volume of 50 ul. Ligated samples were purified with magnetic beads and eluted in 30 ul of 10 mM Tris-HCl pH 7.5. The concentration of each library was checked on a Nanodrop Lite spectrophotometer. The individual sample libraries were pool equally by mass.

The pooled library was amplified by PCR using the P7-pcr primer and a primer specific to all *Alu*Yb8/9 insertions [pooled library template DNA (360 ng), 1X Phusion HF buffer, 0.2 mM dNTPs, 0.4 μM each for primers P7-pcr and *Alu*Yb8_short, Phusion HotStart DNA polymerase (0.6 U), dH_2_0 to 25 ul]. For PCR, the sample was initially denatured for 10 minutes at 98^o^C and then cycled 5 times (98^o^C, 10 sec; 72^o^C, 30 sec) with a final extension at 72^o^C for 5 min. Empirical tests indicated that 5 cycles were optimal to establish sufficient sequencing reads containing *Alu* insertions while limiting duplicate reads to a manageable number.

Following pooled library amplification, the PCR products were size-selected on a 2% agarose mini-gel. An approximately 2 mm gel slice at 635 bp was removed, and products were extracted using a QIAquick gel extraction kit (Qiagen) according to the manufacturer’s specifications. The *Alu*-containing biotinylated fragments were captured from solution using magnetic streptavidin M-270 Dynabeads (Invitrogen) according to the manufacturer’s specifications. These concentrated bead-bound genomic fragments were re-amplified using the P7 primer and a mix of six *Alu*_head primers with the Illumina P5 sequence [concentrated bead-bound fragments (2ul), 1X Phusion HF buffer, 0.2 mM dNTPs, 0.4 μM each for primers P7 and *Alu*_head primers, Phusion HotStart DNA polymerase (1U), dH_2_0 to 25 ul] for 25 cycles [98^o^C, 30 sec; (98^o^C, 10 sec; 65^o^C, 30 sec; 72^o^C, 30 sec) x 25 cycles; 72^o^C, 5 min]. Typically, two independent amplifications were performed to provide ample material for sequencing. The amplified products were separated on a 2% agarose gel and a 2 mm band containing the bulk of the amplification product was excised. The products were removed from the gel using the QIAquick gel extraction method. The library was eluted in 20-30 ul. The size distribution for all finished libraries were checked on an Agilent bioanalyzer. The concentration of each library was determined using a picogreen assay. Libraries were then sequenced on an Illumina HiSeq 2000 using standard methods and version 2 chemistry at the sequencing core facility located in the Huntsman Cancer Institute at the University of Utah.

Supplementary Methods table 1. A listing of primers used for MEscan library construction. Common and long barcode primers (>40 bp) were PAGE purified. AdRC primers were used in early MEscan libraries in multiplexes of 24 samples. Multiplexes were then expanded to 48 samples plus controls using combinations of the original AdRC primers and the u/nAdRC primers. AdRC primers are listed in pairs and each pair, when annealed, form a P7 adaptor used for one sample.

| MEscan primers | |
| --- | --- |
|  | |
| *Common primers for library construction* | |
| PCR_P7 | CAAGCAGAAGACGGCATACGAGA*T |
| PCR_P5 | AATGATACGGCGACCACCGAGATC*T |
| PCR_*Alu*Yb8_Short | /5BiosG/CAGGCCGGACTGCGGA*C |
| PCR_*Alu*Y_Long | /5BiosG/GGATGGTCTCGTTCTCCTGACCTCGTGAT*C |
| PCR_*Alu*Y_Short | /5BiosG/ATGGTCTCGTTCTCCTGACCTCGTGAT*C |
| *Alu*_Head_1 | AATGATACGGCGACCACCGAGATCTACACTCTTTCCCTACACGACGCTCTTCCGATCTRYRAGTGCTGGGATT  ACAGGCGTG*A |
| *Alu*_Head_2 | AATGATACGGCGACCACCGAGATCTACACTCTTTCCCTACACGACGCTCTTCCGATCTYRYAGTGCTGGGATT  ACAGGCGTG*A |
| *Alu*_Head_3 | AATGATACGGCGACCACCGAGATCTACACTCTTTCCCTACACGACGCTCTTCCGATCTRYRYAGTGCTGGGATT  ACAGGCGTG*A |
| *Alu*_Head_4 | AATGATACGGCGACCACCGAGATCTACACTCTTTCCCTACACGACGCTCTTCCGATCTYRYRAGTGCTGGGATT  ACAGGCGTG*A |
| *Alu*_Head_5 | AATGATACGGCGACCACCGAGATCTACACTCTTTCCCTACACGACGCTCTTCCGATCTRYRYRAGTGCTGGGAT  TACAGGCGTG*A |
| *Alu*_Head_6 | AATGATACGGCGACCACCGAGATCTACACTCTTTCCCTACACGACGCTCTTCCGATCTYRYRYAGTGCTGGGAT  TACAGGCGTG*A |
|  |  |
| *Barcoded samples primers* | |
| AdRCP7_t1_t2 | CAAGCAGAAGACGGCATACGAGATCGTGATGTGACTGGAGTTCAGACGTGTGCTCTTCCGATCTACATCG*T |
| AdRCShort_t2 | CGATGTAGATCGGAAGAGCGTCGTG |
| AdRCP7_t2_t3 | CAAGCAGAAGACGGCATACGAGATACATCGGTGACTGGAGTTCAGACGTGTGCTCTTCCGATCTGCCTAA*T |
| AdRCShort_t3 | TTAGGCAGATCGGAAGAGCGTCGTG |
| AdRCP7_t3_t4 | CAAGCAGAAGACGGCATACGAGATGCCTAAGTGACTGGAGTTCAGACGTGTGCTCTTCCGATCTTGGTCA*T |
| AdRCShort_t4 | TGACCAAGATCGGAAGAGCGTCGTG |
| AdRCP7_t4_t5 | CAAGCAGAAGACGGCATACGAGATTGGTCAGTGACTGGAGTTCAGACGTGTGCTCTTCCGATCTCACTGT*T |
| AdRCShort_t5 | ACAGTGAGATCGGAAGAGCGTCGTG |
| AdRCP7_t5_t6 | CAAGCAGAAGACGGCATACGAGATCACTGTGTGACTGGAGTTCAGACGTGTGCTCTTCCGATCTATTGGC*T |
| AdRCShort_t6 | GCCAATAGATCGGAAGAGCGTCGTG |
| AdRCP7_t6_t7 | CAAGCAGAAGACGGCATACGAGATATTGGCGTGACTGGAGTTCAGACGTGTGCTCTTCCGATCTGATCTG*T |
| AdRCShort_t7 | CAGATCAGATCGGAAGAGCGTCGTG |
| AdRCP7_t7_t8 | CAAGCAGAAGACGGCATACGAGATGATCTGGTGACTGGAGTTCAGACGTGTGCTCTTCCGATCTTCAAGT*T |
| AdRCShort_t8 | ACTTGAAGATCGGAAGAGCGTCGTG |
| AdRCP7_t8_t9 | CAAGCAGAAGACGGCATACGAGATTCAAGTGTGACTGGAGTTCAGACGTGTGCTCTTCCGATCTCTGATC*T |
| AdRCShort_t9 | GATCAGAGATCGGAAGAGCGTCGTG |
| AdRCP7_t9_t10 | CAAGCAGAAGACGGCATACGAGATCTGATCGTGACTGGAGTTCAGACGTGTGCTCTTCCGATCTAAGCTA*T |
| AdRCShort_t10 | TAGCTTAGATCGGAAGAGCGTCGTG |
| AdRCP7_t10_t11 | CAAGCAGAAGACGGCATACGAGATAAGCTAGTGACTGGAGTTCAGACGTGTGCTCTTCCGATCTGTAGCC*T |
| AdRCShort_t11 | GGCTACAGATCGGAAGAGCGTCGTG |
| AdRCP7_t11_t12 | CAAGCAGAAGACGGCATACGAGATGTAGCCGTGACTGGAGTTCAGACGTGTGCTCTTCCGATCTTACAAG*T |
| AdRCShort_t12 | CTTGTAAGATCGGAAGAGCGTCGTG |
| AdRCP7_t12_r1 | CAAGCAGAAGACGGCATACGAGATTACAAGGTGACTGGAGTTCAGACGTGTGCTCTTCCGATCTATCACG*T |
| AdRCShort_r1 | CGTGATAGATCGGAAGAGCGTCGTG |
| AdRCP7_r1_r8 | CAAGCAGAAGACGGCATACGAGATATCACGGTGACTGGAGTTCAGACGTGTGCTCTTCCGATCTACTTGA*T |
| AdRCShort_r8 | TCAAGTAGATCGGAAGAGCGTCGTG |
| AdRCP7_r8_r12 | CAAGCAGAAGACGGCATACGAGATACTTGAGTGACTGGAGTTCAGACGTGTGCTCTTCCGATCTCTTGTA*T |
| AdRCShort_r12 | TACAAGAGATCGGAAGAGCGTCGTG |
| AdRCP7_r12_r13 | CAAGCAGAAGACGGCATACGAGATCTTGTAGTGACTGGAGTTCAGACGTGTGCTCTTCCGATCTAGTCAA*T |
| AdRCShort_r13 | TTGACTAGATCGGAAGAGCGTCGTG |
| AdRCP7_r13_r14 | CAAGCAGAAGACGGCATACGAGATAGTCAAGTGACTGGAGTTCAGACGTGTGCTCTTCCGATCTAGTTCC*T |
| AdRCShort_r14 | GGAACTAGATCGGAAGAGCGTCGTG |
| AdRCP7_r14_r16 | CAAGCAGAAGACGGCATACGAGATAGTTCCGTGACTGGAGTTCAGACGTGTGCTCTTCCGATCTCCGTCC*T |
| AdRCShort_r16 | GGACGGAGATCGGAAGAGCGTCGTG |
| AdRCP7_r16_r18 | CAAGCAGAAGACGGCATACGAGATCCGTCCGTGACTGGAGTTCAGACGTGTGCTCTTCCGATCTGTCCGC*T |
| AdRCShort_r18 | GCGGACAGATCGGAAGAGCGTCGTG |
| AdRCP7_r22_r24 | CAAGCAGAAGACGGCATACGAGATCGTACGGTGACTGGAGTTCAGACGTGTGCTCTTCCGATCTGGTAGC*T |
| AdRCShort_r24 | GCTACCAGATCGGAAGAGCGTCGTG |
| AdRCP7_r24_r27 | CAAGCAGAAGACGGCATACGAGATGGTAGCGTGACTGGAGTTCAGACGTGTGCTCTTCCGATCTATTCCT*T |
| AdRCShort_r27 | AGGAATAGATCGGAAGAGCGTCGTG |
| AdRCP7_r27_r33 | CAAGCAGAAGACGGCATACGAGATATTCCTGTGACTGGAGTTCAGACGTGTGCTCTTCCGATCTCAGGCG*T |
| AdRCShort_r33 | CGCCTGAGATCGGAAGAGCGTCGTG |
| AdRCP7_r33_r36 | CAAGCAGAAGACGGCATACGAGATCAGGCGGTGACTGGAGTTCAGACGTGTGCTCTTCCGATCTCCAACA*T |
| AdRCShort_r36 | TGTTGGAGATCGGAAGAGCGTCGTG |
| AdRCP7_r36_r39 | CAAGCAGAAGACGGCATACGAGATCCAACAGTGACTGGAGTTCAGACGTGTGCTCTTCCGATCTCTATAC*T |
| AdRCShort_r39 | GTATAGAGATCGGAAGAGCGTCGTG |
| AdRCP7_r39_r40 | CAAGCAGAAGACGGCATACGAGATCTATACGTGACTGGAGTTCAGACGTGTGCTCTTCCGATCTCTCAGA*T |
| AdRCShort_r40 | TCTGAGAGATCGGAAGAGCGTCGTG |
| AdRCP7_r40_r41 | CAAGCAGAAGACGGCATACGAGATCTCAGAGTGACTGGAGTTCAGACGTGTGCTCTTCCGATCTGACGAC*T |
| AdRCShort_r41 | GTCGTCAGATCGGAAGAGCGTCGTG |
| uAdRCP7_38 | CAAGCAGAAGACGGCATACGAGATGGACGGGTGACTGGAGTTCAGACGTGTGCTCTTCCGATCTTAATCG*T |
| nAdRCShort_38 | CGATTAAGATCGGAAGAGCGTCGTG |
| uAdRCP7_39 | CAAGCAGAAGACGGCATACGAGATTCGGCAGTGACTGGAGTTCAGACGTGTGCTCTTCCGATCTTCATTC*T |
| nAdRCShort_39 | GAATGAAGATCGGAAGAGCGTCGTG |
| uAdRCP7_40 | CAAGCAGAAGACGGCATACGAGATCAAAAGGTGACTGGAGTTCAGACGTGTGCTCTTCCGATCTTGTTGG*T |
| nAdRCShort_40 | CCAACAAGATCGGAAGAGCGTCGTG |
| uAdRCP7_41 | CAAGCAGAAGACGGCATACGAGATATGAGCGTGACTGGAGTTCAGACGTGTGCTCTTCCGATCTTTGACT*T |
| nAdRCShort_41 | AGTCAAAGATCGGAAGAGCGTCGTG |
| uAdRCP7_42 | CAAGCAGAAGACGGCATACGAGATTACAGCGTGACTGGAGTTCAGACGTGTGCTCTTCCGATCTGGACGG*T |
| nAdRCShort_42 | CCGTCCAGATCGGAAGAGCGTCGTG |
| uAdRCP7_43 | CAAGCAGAAGACGGCATACGAGATGTGAAAGTGACTGGAGTTCAGACGTGTGCTCTTCCGATCTATGAGC*T |
| nAdRCShort_43 | GCTCATAGATCGGAAGAGCGTCGTG |
| uAdRCP7_44 | CAAGCAGAAGACGGCATACGAGATATAATAGTGACTGGAGTTCAGACGTGTGCTCTTCCGATCTACACGC*T |
| nAdRCShort_44 | GCGTGTAGATCGGAAGAGCGTCGTG |
| uAdRCP7_45 | CAAGCAGAAGACGGCATACGAGATACCGCCGTGACTGGAGTTCAGACGTGTGCTCTTCCGATCTAGAGAC*T |
| nAdRCShort_45 | GTCTCTAGATCGGAAGAGCGTCGTG |
| uAdRCP7_46 | CAAGCAGAAGACGGCATACGAGATTGGCGCGTGACTGGAGTTCAGACGTGTGCTCTTCCGATCTTCTACG*T |
| nAdRCShort_46 | CGTAGAAGATCGGAAGAGCGTCGTG |
| uAdRCP7_47 | CAAGCAGAAGACGGCATACGAGATCAGTAAGTGACTGGAGTTCAGACGTGTGCTCTTCCGATCTGGCCTT*T |
| nAdRCShort_47 | AAGGCCAGATCGGAAGAGCGTCGTG |
| uAdRCP7_48 | CAAGCAGAAGACGGCATACGAGATATAGAGGTGACTGGAGTTCAGACGTGTGCTCTTCCGATCTGCGCTC*T |
| nAdRCShort_48 | GAGCGCAGATCGGAAGAGCGTCGTG |
| uAdRCP7_49 | CAAGCAGAAGACGGCATACGAGATTGTGTCGTGACTGGAGTTCAGACGTGTGCTCTTCCGATCTAGATGT*T |
| nAdRCShort_49 | ACATCTAGATCGGAAGAGCGTCGTG |
| uAdRCP7_50 | CAAGCAGAAGACGGCATACGAGATGAATTAGTGACTGGAGTTCAGACGTGTGCTCTTCCGATCTATGTAT*T |
| nAdRCShort_50 | ATACATAGATCGGAAGAGCGTCGTG |
| uAdRCP7_51 | CAAGCAGAAGACGGCATACGAGATCTGCGTGTGACTGGAGTTCAGACGTGTGCTCTTCCGATCTGTGGTG*T |
| nAdRCShort_51 | CACCACAGATCGGAAGAGCGTCGTG |
| uAdRCP7_52 | CAAGCAGAAGACGGCATACGAGATGATTATGTGACTGGAGTTCAGACGTGTGCTCTTCCGATCTTGGCGC*T |
| nAdRCShort_52 | GCGCCAAGATCGGAAGAGCGTCGTG |
| uAdRCP7_53 | CAAGCAGAAGACGGCATACGAGATGCTCGTGTGACTGGAGTTCAGACGTGTGCTCTTCCGATCTAGCGCT*T |
| nAdRCShort_53 | AGCGCTAGATCGGAAGAGCGTCGTG |
| uAdRCP7_54 | CAAGCAGAAGACGGCATACGAGATTGACTTGTGACTGGAGTTCAGACGTGTGCTCTTCCGATCTATGGCA*T |
| nAdRCShort_54 | TGCCATAGATCGGAAGAGCGTCGTG |
| uAdRCP7_55 | CAAGCAGAAGACGGCATACGAGATCTACCGGTGACTGGAGTTCAGACGTGTGCTCTTCCGATCTCTGCAG*T |
| nAdRCShort_55 | CTGCAGAGATCGGAAGAGCGTCGTG |
| uAdRCP7_56 | CAAGCAGAAGACGGCATACGAGATGGCGTAGTGACTGGAGTTCAGACGTGTGCTCTTCCGATCTTATGCT*T |
| nAdRCShort_56 | AGCATAAGATCGGAAGAGCGTCGTG |
| uAdRCP7_57 | CAAGCAGAAGACGGCATACGAGATAATGCAGTGACTGGAGTTCAGACGTGTGCTCTTCCGATCTGGTGAG*T |
| nAdRCShort_57 | CTCACCAGATCGGAAGAGCGTCGTG |
| uAdRCP7_58 | CAAGCAGAAGACGGCATACGAGATCAACTCGTGACTGGAGTTCAGACGTGTGCTCTTCCGATCTTCTCAC*T |
| nAdRCShort_58 | GTGAGAAGATCGGAAGAGCGTCGTG |
| uAdRCP7_59 | CAAGCAGAAGACGGCATACGAGATAGCTAGGTGACTGGAGTTCAGACGTGTGCTCTTCCGATCTCAACTC*T |
| nAdRCShort_59 | GAGTTGAGATCGGAAGAGCGTCGTG |
| uAdRCP7_60 | CAAGCAGAAGACGGCATACGAGATGAGGTTGTGACTGGAGTTCAGACGTGTGCTCTTCCGATCTTCCGTT*T |
| nAdRCShort_60 | AACGGAAGATCGGAAGAGCGTCGTG |
| uAdRCP7_61 | CAAGCAGAAGACGGCATACGAGATGGCCATGTGACTGGAGTTCAGACGTGTGCTCTTCCGATCTTAGCAT*T |
| nAdRCShort_61 | ATGCTAAGATCGGAAGAGCGTCGTG |

*phosphorothioate bond.
