# Supplementary material for: Discovery of rare, diagnostic AluYb8/9 elements in diverse human populations
Source: Mob DNA. 2017 Jul 27;8:9. doi: 10.1186/s13100-017-0093-0 (PMC5531096; doi:10.1186/s13100-017-0093-0)
Supplement: Supplementary file 3 — This file contains Figures S1-S5 and figure legends. (PDF 4383 kb) [file 13100_2017_93_MOESM3_ESM.pdf]

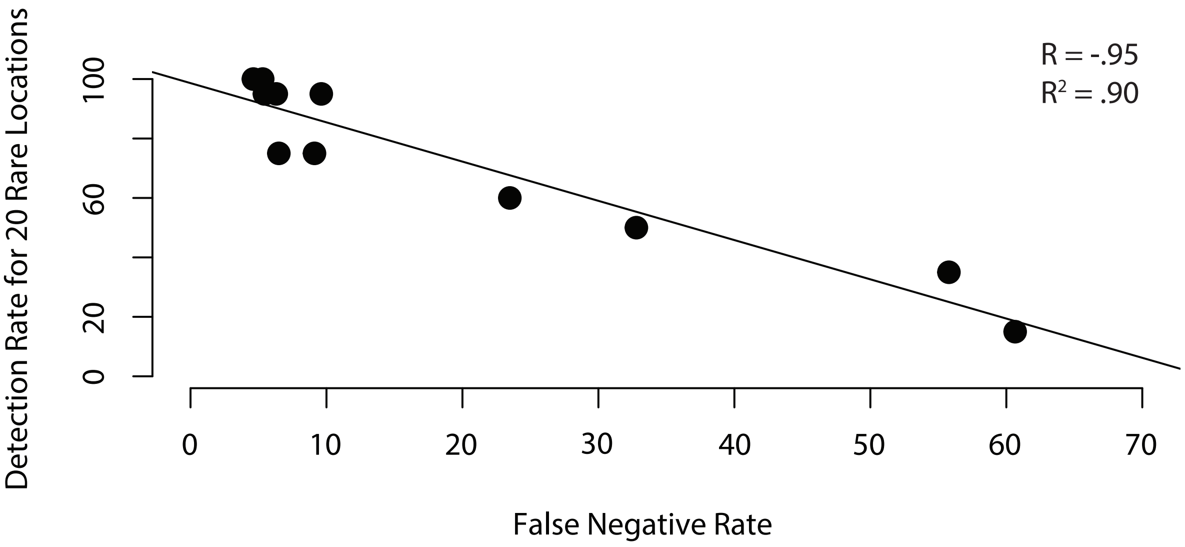

Figure S1: Relationship between the false negative rate and detection rate for rare locations. We optimized a set of 1601 "Good" *Alu*Yb8/9 elements from our previous dataset based on these criteria: not located within segmental duplications, highly likely to be fixed in the human genome, and should be easily captured by ME-Scan. For each replicate, we divided the number of missed Good *Alu* elements by the total Good *Alu* elements (1601) to find the false negative rate per replicate. We also determined the number of rare identified singletons and doubletons (n=20) per replicate. This graph shows the relationship between the false negative rate and the detection rate.

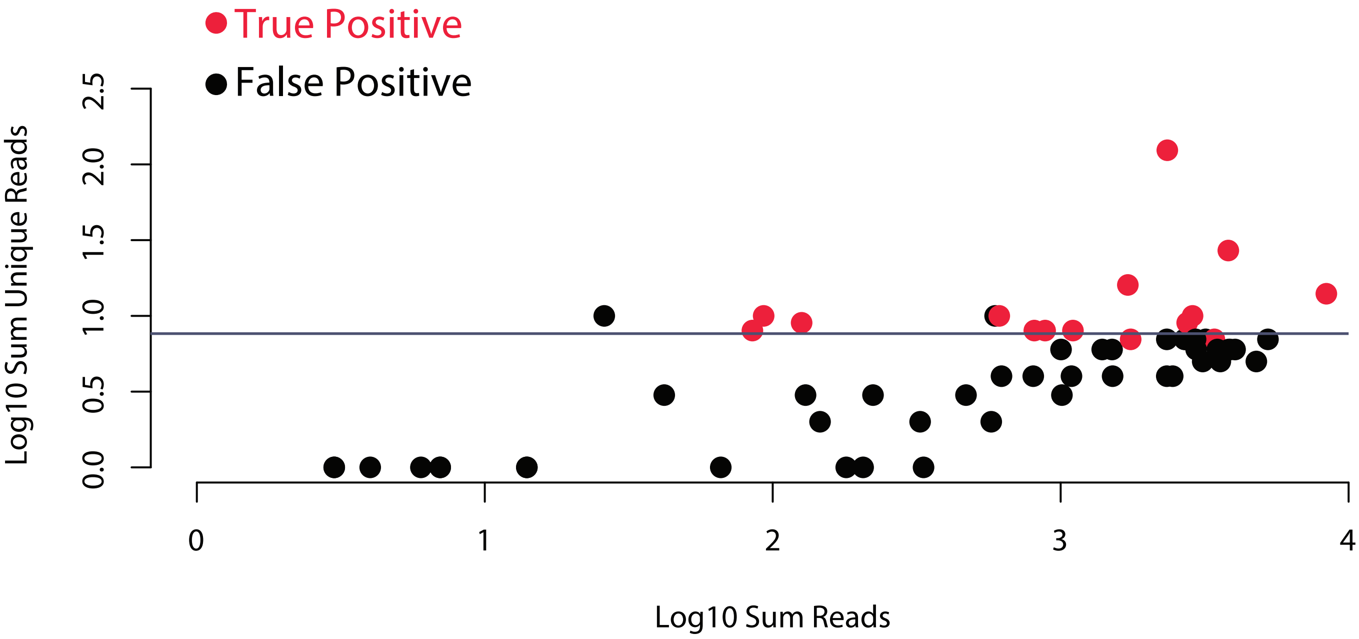

Figure S2: True positive analysis of singletons in dataset. The line indicates the threshold (8 unique reads) cutoff. Locations were randomly selected primers on a log10 read, log10 unique reads scale. We then attempted to even out the coverage, as the vast majority of singletons only had one read of support. This graph represents PCR validation of 60 *Alu* elements found in singletons in the dataset.

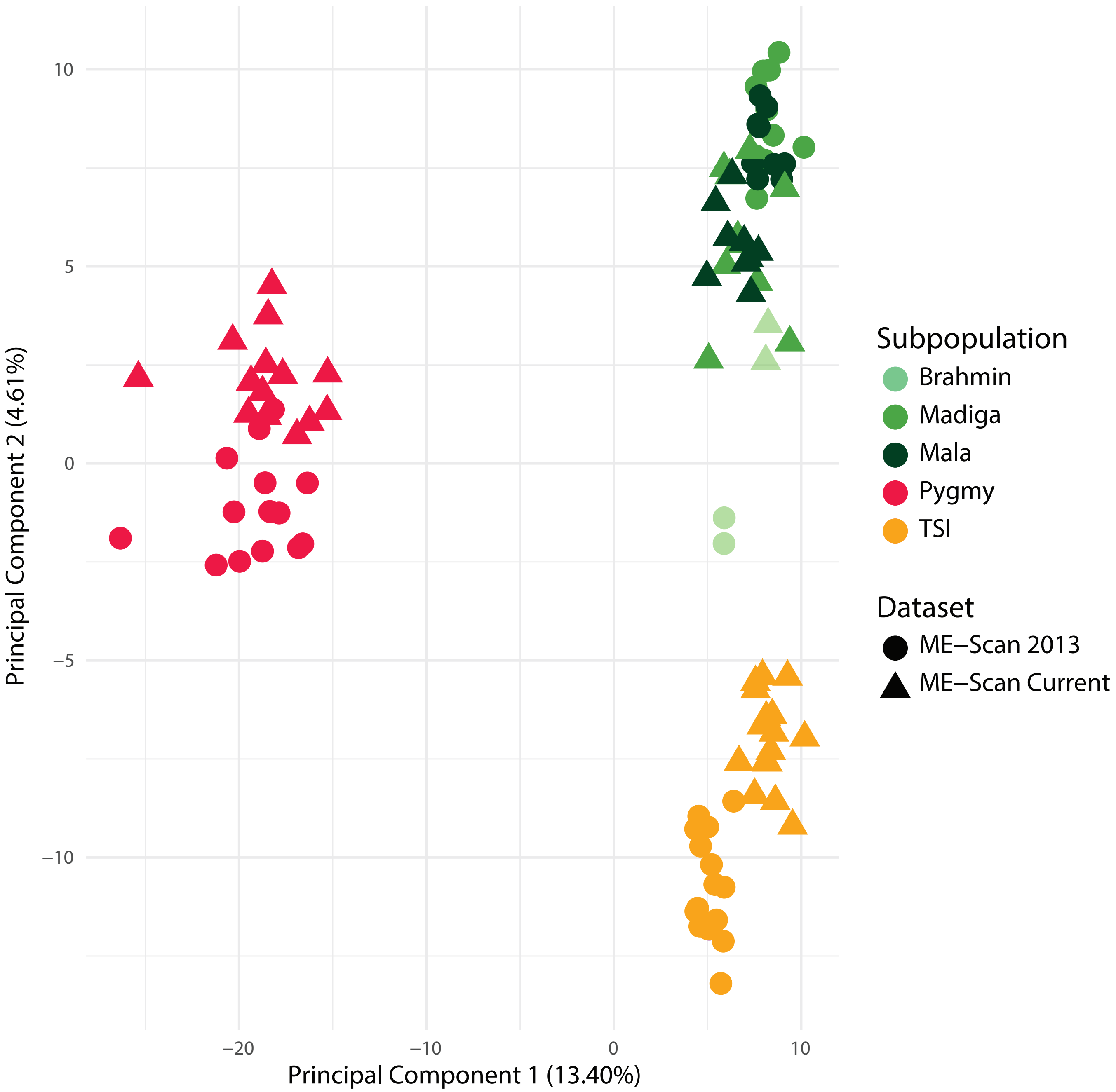

Figure S3: PCA of ME-Scan datasets. We compared the population structure of 51 individuals with <10% false negative rate in this dataset and 100,000 read sets from Witherspoon et al., (2013). Good *Alu* loci and loci with minor allele frequencies less than 5% or greater than 95% (all samples) were removed, which resulted in 1036 shared loci.

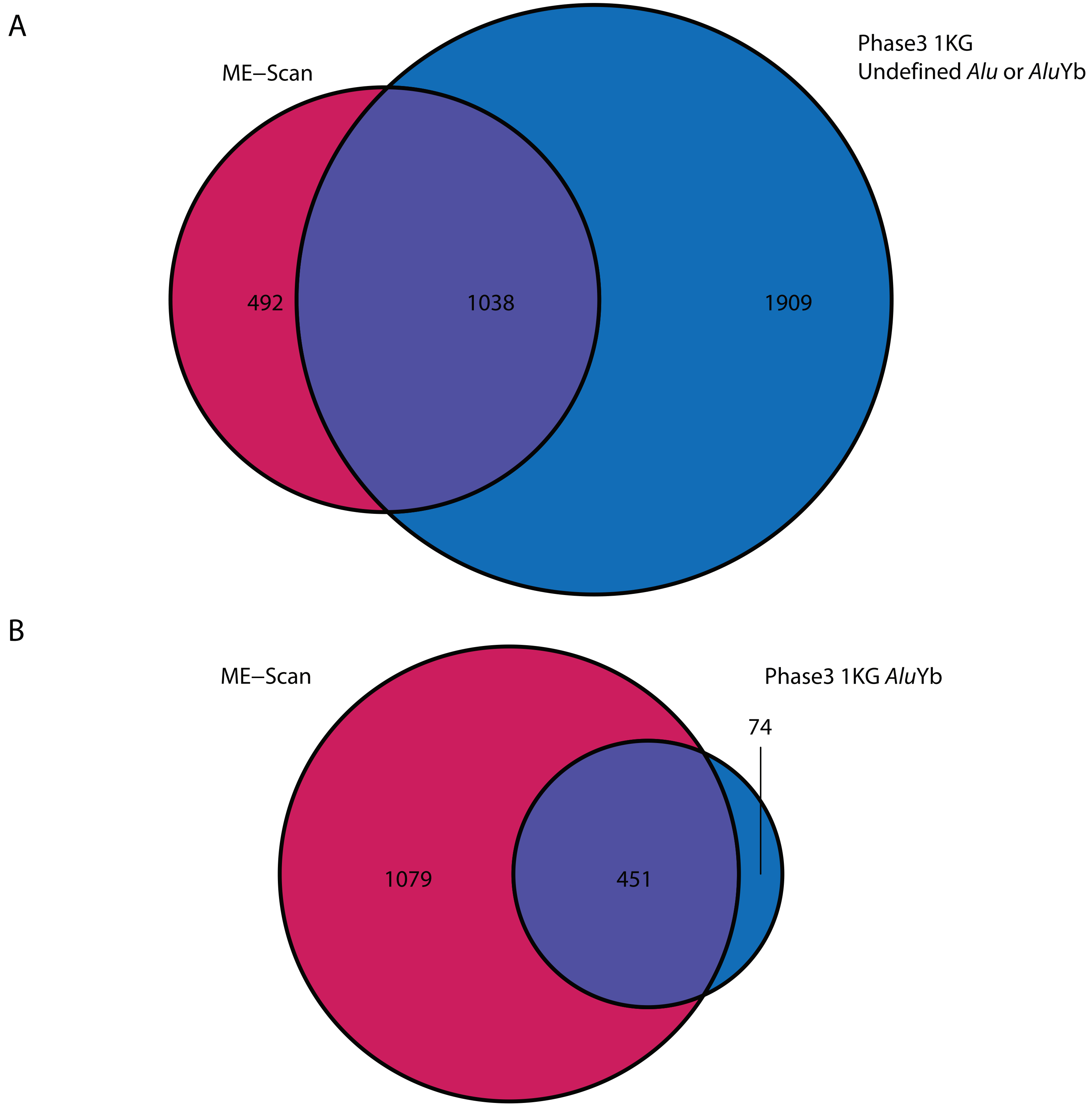

Figure S4: Venn Diagram of non-reference elements from ME-Scan and a set of elements from Phase3 in 1KG individuals.

A. Venn Diagram of non-reference elements from ME-Scan and undefined *Alu* or *Alu*Yb elements from Phase3 in 1KG individuals. The 4,670 *Alu* elements in 41 1KG individuals were filtered for either an *Alu* element with an undefined subfamily, or an *Alu*Yb element. This resulted in 2,947 *Alu* elements.

B. Venn Diagram of non-reference elements from ME-Scan and *Alu*Yb elements from Phase3 in 1KG individuals. The 4,670 *Alu* elements in 41 1KG individuals were filtered for AluYb elements. This resulted in 525 *Alu*Yb elements. 86% of the elements from Phase3 1KG were also detected in the individuals by ME-Scan.

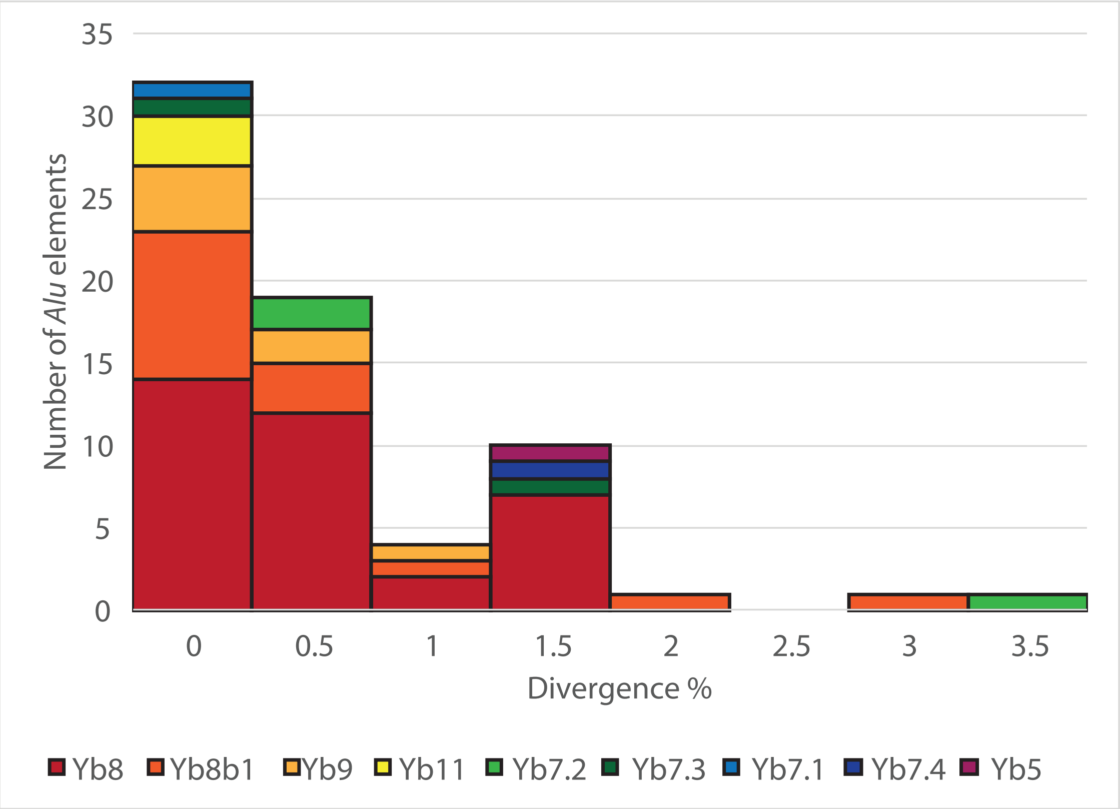

Figure S5: Divergence of subfamily consensus for 68 *Alu* elements based on BLAST+. The entire length of each element was compared to the proper consensus subfamily using BLAST+. >95% of *Alu* elements had a divergence of <2% and >45% of elements did not diverge from the consensus sequence.
