# Supplementary material for: Discovery of rare, diagnostic AluYb8/9 elements in diverse human populations
Source: Mob DNA. 2017 Jul 27;8:9. doi: 10.1186/s13100-017-0093-0 (PMC5531096; doi:10.1186/s13100-017-0093-0)
Supplement: Supplementary file 4 — FASTA sequences of 68 Alu elements. This file contains high-quality sequence from Sanger sequencing of 68 Alu elements. The nucleotides are color-coded for Alu, TSD, A and B boxes, SRP9/14 sites, and pol III termination signals (DOCX 34 kb) [file 13100_2017_93_MOESM4_ESM.docx]

FASTA sequences of 68 *Alu* elements

**Key:**

Genomic flank

TSD

*Alu* sequence

A (TGGCTCACGCC) and B (GATCGAGACC) Box promoter elements

SRP9/14 sites (GCCTGTAATC and GCCTGTAGTC) inside *Alu* element

RNA Polymerase III Termination signal

>*Alu*Yb8_element_hg19_ Chr1:22,901,909_population_analysis

GGAAATGGGGAAGCTATGTGCAAATCATGTAGCTGATAAGGGATTAATATCCAGAATACATAAAGAATTCCCACAACTCGACAACAACAGAAAAGAAACAGTCCAATTAGAAAATGGGCGGCCGGGCGCGGTGGCTCACGCCTGTAATCCCAGCACTTTGGGAGGCCGAGGCGGGTGGATCATGAGGTCAGGAGATCGAGACCATCCTGGCTAACAAGGTGAAACCCCGTCTCTACTAAAAATACAAAAAATTAGCCGGGCGCGGTGGCGGGCGCCTGTAGTCCCAGCTACTCGGGAGGCTGAGGCAGGAGAATGGCGTGAACCCGGGAAGCGGAGCTTGCAGTGAGCCGAGATTGCGCCACTGCGGTCCGCAGTCCGGCCTGGGCGACAGAGCGAGACTCCGTCTCAAAAAAAAAAAAAAAAAAAAAAAAAAAAAAAAAAAAGAAAATGGGCAAAGGACTTGAACAGACATTCTCTAAAGAAAA

>*Alu*Yb8_element_hg19 _Chr1:111,532,094_replicate_analysis

AGCATTCTGGGCTCTGAAAAAATGAGCAAAGGATCTGAATAAACATTTCTTAAAAAAAGGGCCGGGCGCGGTGGCTCACGCCTGTAATCCCAGCACTTTGGGAGGCCGAGGCGGGTGGATCATGAGGTCAGGAGATCGAGACCATCCTGGCTAACAAGGTGAAACCCCGTCTCTACTAAAAATACAAAAAAATTAGCCGGGCTCGGTGGCGGGCGCCTGTAGTCCCAGCTACTCGGGAGGCTGAGGCAGGAGAATGGCGTGAACCCGGGAAGCGGAGCTTGCAGTGAGCCGAGATTGCGCCACTGCAGTCCGCAGTCCGGCCTGGGCGACAGAGCGAGACTCCGTCTCAAAAAAAAAAAAAAAAAAAAAAAAAAAAAAAAAAAAAAAAGACATATAACAACAGATAATAAAAA

>*Alu*Yb8_element_hg19_Chr1:167,220,246_replicate_analysis

TTTGCTGGAATTCTAAAAGCAAATTAAGACATTATTTTAGTAGTTTCAGAATTGTGTTCCTCAAGAGAACAGTAAAGATTTAAGTAGGCCGGGCGCGGTGGCTCACGCCTGTAATCCCAGCACTTTGGGAGGCCGAGGCGGGTGGATCATGAGGTCAGGAGATCGAGACCATCCTGGCTAACAAGGTGAAACCCCGTCTCTACTAAAAATACAAAAAATTAGCCGGGCGCGGTGGCGGGCGCCTGTAGTCCCAGCTACTCGGGAGGCTGAGGCAGGAGAATGGCGTGAACCCGGGAAGCGGAGCTTGCAGTGAGCCGAGATTGCGCCACTGCAGTCCGCAGTCCGGCCTGGGCGACAGAGCGAGACTCCGTCTCAAAAAAAAAAAAAAAAAAAAAAAAAAAAAAAAAAAAAAAAAAAAAAAAAAAGATTTAAGTAACAACAGTTTTGGTTAATAACAGTGAAATTTTCTTTTTGTGAGGTGTATTATAAGAACATCTCATATGAGTAGAACATGAGGTATTTTAGCTTTAAAGCTAATCTTGGCCAAGTG

>*Alu*Yb8b1_element_hg19_Chr2:39,627,428_replicate_analysis

ATGTGAAAGCTTCGATGCATGGATGGTAGAGTCAAGACTGGAGACTGGGCCGGGCGCGGTGGCTCACGCCTGTAATCCCAGCACTTTGGGAGGCCGAGGCGGGTGGATCATGAGGTCAGGAGATCGAGACCATCCTGGCTAACAAGGTGAAACCCCGTCTCTACTAAAAATACAAAAAATTAGCCGGGCGCGGTGGCGGGCGCCTGTAGTCCCAGCTACTCGGGAGGCTGAGGCAGGAGAATGGCGTGAACCCGGGAAGCGGAGCTTGCAGTGAGCCGAGATTGCGCCACTGCAGTCCGCAGTCCGACCTGGGCGACAGAGCGAGACTCCGTCTCAAAAAAAAAAAAAAAAAAAAAAAAAAAAAAAAAAAAAAGACTGGAGACTGTAG

>*Alu*Yb8_element_hg19_Chr2:109,085,309_population_analysis

GGTGGATTGACCACATGCGTAACTATCTCCTGACCTTTTCAAAACACACTGAAGTGAGAATAAGAGATTTTAAAAGGTAGAACACATGGCCGGGCGCGGTGGCTCACGCCTGTAATCCCAGCACTTTGGGAGGCCGAGGCGGGTGGATCATGAGGTCAGGAGATCGAGACCATCCTGGCTAACAAGGTGAAACCCCGTCTCTACTAAAAATACAAAAAATTAGCCGGGCGCGGTGGCGGGCGCCTGTAGTCCCAGCTACTCGGGAGGCTGAGGCAGGAGAATGGCGTGAACCCGGGAAGCGGAGCTTGCAGTGAGCCGAGATTGCGCCACTGCAGTCCGCAGTCTGGCCTGGGCGACAGAGCGAGACTCCGTCTAAAAAAAAAAAAAAAAAAAAGGTAGAACACATTGCGCAAAGAGAAAAGGAGAAAAGATATAAGGATAAGAGAAATTTCAACACATCTTTGGAAAACTGAATTACGGAGGAGGATGAAC

>*Alu*Yb8_element_hg19_Chr2:140,290,059_singleton_analysis

AGAGGTTAGGAAGATCAGGAGATAAATTTTCAAGGAAAAAAATCTGAAAAAAGATTGGCATTTCCACAAATGTGTGAAAGGGAATGCAGACAGATAAACCTAAAGCTAATATCACCAAACACCAATATCCCTGTCAAGCTTTAAGGTCCCAGTGACTTTACTAGGGCAATTTCAATTTTAGCTTTTATGCTCCTGTTCTTACTACAGTTAGTAAAAAGCAAAGGACAGGAGTCAAAACCAGAAATGATCATTTGAAATAAGAACCAAAGCAGACACCTCTGTTGGTCTCTTTTTCTAGCTCTACTTTTCAGGAATTCTGTCTCCAACTATATGACACACTTACTTTTAGAAAGAAACTGCTTGGCCGGGCGCGGTGGCTCACGCCTGTAATCCCAGCACTTTGGGAGGCCGAGGCGAGTGGATCATGAGGTCAGGAGATCGAGACCATCCTGGCTAACAAGGTGAAACCCCGTCTCTACTAAAAATACAAAAAATTAGCCGGGCGCGGTGGCGGGCGCCTGTAGTCCCAGCTACTCGGGAGGCTGAGGCAGGAGAATGGCGTGAACCCGGGAAGCGGAGCTTGCAGTGAGCCGAGATTGCGCCACTGCAGTCCGCAGTCCGGCCTGGGCGACAGAGCGAGACTCCGTCTCAAAAAAAAAAAAAAAAAAAAAAAAAAAAAAAAAAAAAAAGAAACTGCTTTGAGCATTCAAAAAAGGAGGTGATGATGTCAAATGACTAAACATTTCCCAAGTTCTAAACTTTAAAGTCAGTCCAATACTTTCTGTTGAAGCTTTAAAGTATTGATGTTAAG

>*Alu*Yb11_element_hg19_Chr2:143,889,252_singleton_analysis

ATAGACCCTTCCATTCACTGGCTCCTTCCATTCAACTGAACTTTTAAAATGTACTCATCCCATTTTCCAGCACTTAGGGAATCTAATAAAAATAATCCACGCTTGGCCGGGCGCGGTGGCTCACGCCTGTAATCCCAGCACTTTGGGAGGCCGAGGCGGGTGGATCATGAGGTCAGGAGATCGAGACCATCCTGGCTAACAAGGTGAAACCCCGTCTCTACTAAAAATACAAAAAATTAGCCGGGCGCGGTGGCGGGCGCCTGTAGTCCCAGCTACTGGGGAGGCTGAGGCAGGAGAATGGCGTTGAACCCGGGAAGCGGAGCTTGCAGTGAGCCGAGATTGCGCCACTGCAGTCCGCAGTCCAGCCTGGGCGACAGAGCGAGACTCCGTCTCAAAAAAAAAAAAAAAAAAAAAAAAAAAAAAAAATAATCCACGCTTATAGGAGAAGGCTACAGAGCTTTGACTTCCTAGCCAGGCCTCCCTTTCTCCTCCCAGTGGAATTTTAATATGTTTCCTCAGCAGACAGGGCCCAAAGGATTAGCTAAATGT

>*Alu*Yb7.3_element_hg19_Chr2:180,333,266_doubleton_analysis

TTCCTCAGGGAGTAGGTTCAGTAGAGATTCAAAAGCTGGGAGTAAGATTAAAAACTAGGCCGGGCGCGGTGGCTCACGCCTGTAATCCCAGCACTTTGGGAGGCCGAGGCGGGTGGATCACGAGGTCAGGAGATCGAGACCATCCTGGCTAACAAGGTGAAACCCCGTCTCTACTAAAAATACAAAAAATTAGCCGGGCGCGGTGGCGGGCGCCTGTAGTCCCAGCTACTCGGGAGGCTGAGGCAGGAGAATGGCGTGAACCCGGGAAGCGGAGCTTGCAGTGAGCCGAGATTGCGCCACTGCAGTCCGCAGTCCGGCCTGGGCGACAGAGCGAGACTCCGTCTCAAAAAAAAAAAAAAAAAAAAAAAAAAAAGATTAAAAACTAATTTTCTAGGTGAAATGAGAAAAGGTTCTTTTTCTTTGAAATGAATGGCTCTTTATAGAGGAAACAGA

>*Alu*Yb9_element_hg19_Chr2:185,525,222_singleton_analysis

ATAATTCTCTTATTGGCAGGTGTTTAGATTGTCTCACACAGTTTTCTCTAGATATCCCAAGTGTAGCAATTCCTCCTTCATATTCCAGATTTTGTGTATGTGTTTTTTCATAGTAGACTACGTTTCTAGAAGGCAAACACAAATGGCTCTTTGAATCTTCACAGTGCTTAGAAGGACATTTGACATAGTATGAGAGATTAATAAATGATTTTTTTAATTGAAGAATTGTGTTCAGGACTCAGTCCTTTCATTAACTTTAGTGAGTATGGGCACAGAAGACAAGAGGAGAGAAGACAGGCAGTGGGCCGGGCGCGGTGGCTCACGCCTGTAATCCCAGCACTTTGGGAGGCCGAGGCGGGTGGATCATGAGGTCAGGAGATCGAGACCATCCTGGCTAACAAGGTGAAACCCCGTCTCTACTAAAAATACAAAAAATTAGCCGGGCGCGGTGGCGGGCGCCTGTAGTCCCAGCTACTGGGGAGGCTGAGGCAGGAGAATGGCGTGAACCCGGGAAGCGGAGCTTGCAGTGAGCCGAGATTGCGCCACTGCAGTCCGCAGTCCGGCCTGGGCGACAGAGCGAGACTCCGTCTCAAAAAAAAAAAAAAAAAAAAAAAAAAAAAAAGACAGGCAGTGGTGGGGTGCATGCAAAGTTTTCTCCAAATATTTGAAATATCTGTTTCTGAAATAATTTATTGACAAGCTTTCTGTTTTATTTAGAGAATCTAAG

>*Alu*Yb8_element_hg19_Chr2:226,310,093_replicate_analysis

CCAAAATACCACGTTCACCTAAAGTATGTAAAAATTTTCCTAAAGTTTTTTAGGAACTTATGCTAAAGTACATAAAATTATGTACTTTATGTACTTTTCCTAAAGTTTTTTAGGAAATTATGCTAAAGTACATAAAAATTTTCCTCTTTTAATTTTTTAAAGAATAACAAAGGGCCGGGCGCGGTGGCTCACGCCTGTAATCCCAGCACTTTGGGAGGCCGAGGCGGGTGGATCATGAGGTCAGGAGATCGAGACCATCCTGGCTAACAAGGTGAAACCCCGTCTCTACTAAAAATACAAAAAATTAGCCGGGCGCGGTGGCGGGCGCCTGTAGTCCCAGCTACTCGGGAGGCTGAGGCAGGAGAATGGCGTGAACCCGGGAAGCGGAGCTTGCAGTGAGCCGAGATTGCGCCACTGCAGTCCGCAGTCCGGCCTGGGCGACAGAGCGAGACTCCGTCTCAAAAAAAAAAAAAAAAAAAAAAAAAAAAAAAAAAAAAAAAAAAAAGAATAACAAAGTATCAATTGAACATAAAAGTGGCTCTCACCCACTAATATTTCTGATGCTTAATTTGAATTTATATAAAATCAGTAAGGCAG

>*Alu*Yb8_element_hg19_Chr2:237,994,278_UTR_analysis

TCAGAGCAGGGAACTCACGAACGTTTCGTGAGCACCTACGGTATGCAGGGTACGGTGCGGAGCCCTAGCGTCACTGACGACCGGGACAGCCGAGCAGCTGCAGGATCCGTCGTGTTCCCCAGCAATTTTAATATTTAAAAATTCGTTACTGTTGGCCGGGCGCGGTGGCTCACGCCTGTAATCCCAGCACTTTGGGAGGCCGAGGCGGGTGGATCATGAGGTCAGGAGATCGAGACCATCCTGGCTAACAAGGTGAAACCCCGTCTCTACTAAAAATACAAAAAATTAGCCGGGCGCGGTGGCGGGCGCCTGTAGTCCCAGCTACTCGGGAGGCTGAGGCAGGAGAATGGCGTGAACCCGGGAAGCGGAGCTTGCAGTGAGCCGAGATTGCGCCACTGCAGTCCGCAGTCCGGCCTGGGCGACAGAGCGAGACTCCGTCTCAAAAAAAAAAAAAAAAAAAATTCGTTACTGTTGCTGTTACTTGTTTTCTATGTATTGGATGTCTTCGTGAAGAACCCTCAAAAGTGCAACGAACTCCTCCCTGCCAGAGGGCGGCCGCGCGCTCTGAGTACAGCTTCCCGCGGAGCCGGCCAGGTCCTCCAGGGCACCGAGAAAGCCGGCCAGAACGGCGGCGCCCTATCCCGGCCGCAGCGATGTCTGACGGCGCCCCGGAACTGACGGTCTGGTACGCAGGGGCGCTCGGCGGCAACGGCGGCTTTAAACGTCATCGCGGGCGCGACGCCTGAGGGACAGTCTGGGGTTTGGCTGTCCGGACGGTGCAGCGGCGAGGCCGGCCGCGAAGATGCCAGTGGCGGTGATGGCGGAAAGCGCCTTTAGTTTCAAAAAGTTGCTGGATCAGTGCGAGAACCAGGAGCTCGAGGTAACCCTTG

>*Alu*Yb8_element_hg19_Chr2:242,302,150_population_analysis

CAGCTCCACATCCATGCATTTTAAAATACATTTATAGTGGTTATCATTGGGAGGGGTAAGAGGATTGATTTTTATTTCCTTCTTCCTAATTTTCTGTATTTGTCAAATCACCTACAGTGACGAGTATACATTATCTTTGAAGTCAGATGGTTTCTCTGCAGAATCCTGCAGAAGTACTACCAGCTTTAAAAGTCTTAACTGGGCCGGGCGCGGTGGCTCACGCCTGTAATCCCAGCACTTTGGGAGGCCGAGGCGGGTGGATCATGAGGTCAGGAGATCGAGACCATCCTGGCTAACAAGGTGAAACCCCGTCTCTACTAAAAATACAAAAAATTAGCCGGGCGCGGTGGCGGGCGCCTGTGGTCCCAGCTACTCGGGAGGCTGAGGCAGGAGAATGGCGTGAACCCGGGAAGCGGAGCTTGCAGTGAGCCGAGATTGCGCCACTGCAGTCCGCAGTCCGGCCTGGGCGACAGAGCGAGACTCCGTCTCAAAAAAAAAAAAAAAAAAAAAAAAAAAGTCTAACTGATAGGGGCTACTACATTG

>*Alu*Yb9_element_hg19_Chr3:56,887,230_singleton_analysis

TTTCAATTCAGCACCCTCCTGTAGTACCTGAGCATGGGGTACAGAGGTAACAGAACAGAGGACATGCATTGTTAAAGAAAAATCTGGGCCGGGCGCGGTGGCTCACGCCTGTAATCCCAGCACTTTGGGAGGCCGAGGCGGGTGGATCATGAGGTCAGGAGATCGAGACCATCCTGGCTAACAAGGTGAAACCCCGTCTCTACTAAAAATACAAAAAATTAGCCGGGCGCGGTGGCGGGCGCCTGTAGTCCCAGCTACTGGGGAGGCTGAGGCAGGAGAATGGCGTGAACCCGGGAAGCGGAGCTTGCAGTGAGCCGAGATTGCGCCACTGCAGTCCGCAGTCCGGCCTGGGCGACAGAGCGAGACTCCGTCTCAAAAAAAAAAAAAAAAAAAAAAAAAAAAGAAAAATCTGGGGTGGTGACAAGCCAAAAGTCTACCAGCCAAAAGGTCGAGAGATTGTGTAGAAGGAAGCTCACATCT

>*Alu*Yb8_element_hg19_Chr3:68,425,109_replicate_analysis

GATGGTCAATGAAAAACATCACTTAATTAGGAGATGGTTCTTAAAAACTCTCTGGCTGGGCCGGGCGCGGTGGCTCACGCCTGTAATCCCAGCACTTTGGGAGGCCGAGGCGGGTGGATCATGAGGTCAGGAGATCGAGACCATCCTGGCTAACAAGGTGAAACCCCGTCTCTACTAAAAATACAAAAAATTAGCCGGGCGCGGTGGCGGGCGCCTGTAGTCCCAGCTACTCGGGAGGCTGAGGCAGGAGAATGGCGTGAACCCAGGAAGTGGAGCTTGCAGTGAGCCGAGATTGCGCCACTGCAGTCCGCAGTCTGGCCTGGGCGACAGAGCGAGACTCCGTCTCAAAAAAAAAAAAAAAAAAAAAAAAAACTCTCTGGCTGTGCCTTTCGAAGGCTTTAAAAATATATAAATACTTTGGCTGCTTCTTTAGAGAT

>*Alu*Yb8_element_hg19_Chr3:72,058,748_population_analysis

CCAGAAAAGGAGGGTGAGGGGTAGGAAAGTCAGCCAGTTAGCCAGATGTTTTTGCTTCCAAATTTTATACTAATTCATTCAGTAATTTGTTCCTCAAAACCTGCACATGTGCAAGGTTCTCTGCTAGGAATGTTGGAAGGTAAATTCAGAGAGGTGACCTCAAATATCATTCTAACTGAAGTTGTGGTATGTGTAACCCATTTAAAAAATAAGAAAATATAAGAGTCATTTAAGGCCGGGCGCGGTGGCTCACGCCTGTAATCCCAGCACTTTGGGAGGCCGAGGCGGGTGGATCATGAGGTCAGGAGATCGAGACCATCCTGGCTAACAAGGTGAAACCCCGTCTCTACTAAAAATACAAAAAATTAGCCGGGCGCGGTGGCGGGCGCCTGTAGTCCCAGCTACTCGGGAGGCTGAGGCAGGAGAATGGCGTGAACCCGGGAAGCGGAGCTTGCAGTGAGCCGAGATTGCGCCACTGCAGTCCGCAGTCCGGCCTGGGCGACAGAGCGAGACTCCGTCTCAAAAAAAAAAAAAAAAAAAAAAAAAAAGAGTCATTTAAGTAAGCTACAATATAAAGCAGAGAAAGATAAATACCTACTGG

>*Alu*Yb8_element_hg19_Chr3:164,227,584_singleton_analysis

GCTCTCAGTCTTCCAGAACCTTTTATGTTAGTATATTCTTTCCATGATTAAATATCTACTAATTTAGATTAGCTTATGGTTACTTAAACATAGTAAAATCTTCAAAATATCTCATCCAAAATTGCCTTCAGCATGTAAGTACAAAATGATAATAACTGATTTTAAAATAAATTACTAGTAAAGTTAAAACTGGTCAATGCTGTTTTGTCTTATTAAATGCAAATTCATTTTGATCCTCTTTTTCTCTAACTATTCACTGCTCTTACATTCTATCAATCGTAAAATTCATAAAGAAAAATAATGAAGTGGGGCCGGGCGCGGTGGCTCACGCCTGTAATCCCAGCACTTTGGGAGGCCGAGGCGGGTGGATCATGAGGTCAGGAGATCGAGACCATCCTGGCTAACAAAGTGAAACCCCGTCTCTACTAAAAATACAAAAAATTAGCCGGGCGCGGTGGCGGGCGCCTGTAGTCCCAGCTACTCGGGAGGCTGAGGCAGGAGAATGGCGTGAACCCAGGAAGTGGAGCTTGCAGTGAGCCGAGATTGCGCCACTGCAGTCCGCAGTCTGGCCTGGGCGACAGAGCGAGACTCCGTCTCAAAAAAAAAAAAAAAAAAAAAAAAAAAAAAAAAAAAAAAAAAAAAAAAAAGAAAAATAATGAAGTGATATGAATAAGAAATGAGATATTTATCTTTTTATATAACTTTCAAAAGTGAAAGCCCAATAATATTGAATTTCTCCTAAAGTATAACTTTTCTG

>*Alu*Yb8_element_hg19_Chr3:170,511,204_replicate_analysis

TGGCACTTCATCTCCATGGTCTTCCCCCAAACTCATAATCCCAGCCTAATCATAAGAAAAACATTAGGCAAATTCTAATTGAGGGACATCTACAAAAAGCCTGACCAGTATTCCTCAGAGTTATAAAGGTCATAAAAAACAAGAAGTGTGGCCGGGCGCGGTGGCTCACGCCTGTAATCCCAGCACTTTGGGAGGCCGAGGCGGGTGGATCATGAGGTCAGGAGATCGAGACCATCCTGGCTAACAAGGTGAAACCCCGTCTCTACTAAAAATACAAAAAATTAGCCGGGCGCGGTGGCGGGCGCCTGTAGTCCCAGCTACTCGGGAGGCTGAGGCAGGAGAATGGCGTGAACCCGGGAAGCGGAGCTTGCAGTGAGCCGAGATTGCGCCACTGCAGTCCGCAGTCCGGCCTGGGCGACAGAGCGAGACTCCGTCTCAAAAAAAAAAAAAAAAAAAAAAAAAAAAAAAAAAAAAAAAAAAAAAAAAAAAAAAAAAACAAGAAGTGTTTGAGAAACCGTCGTAATTGAGGTGTCTATGGAGACATGACTACTAAATGCAA

>*Alu*Yb8_element_hg19_Chr3:176,228,597_replicate_analysis

GCCATTGTTTAGCCACCAAATCAAGTTCCTTATTTAGGTAACCCACTGGTTGTTGAGCTGGTCCTTGGGTCTGTGTTAAAACTCCCAGGGCCGGCCGGGCGCGGTGGCTCACGCCTGTAATCCCAGCACTTTGGGAGGCCGAGGCGGGTGGATCATGAGGTCAGGAGATCGAGACCATCCTGGCTAACAAGGTGAAACCCCGTCTCTACTAAAAAATAAAAAATACAAAAAATTAGCCGGGCGTGGTGGCGGGCGCCTGTAGTCCCAGCTACTCGGGAGGCTGAGGCAGGAGAATGGCGTGAACCCAGGAAGCGGAGCTTGCAGTGAGCCGAGATTGCGCCACTGCAGTCCGCAGTCCGGCCTGGGCGACAGAGCGAGACTCCGTCTCAAAAAAAAAAAAAAAAAAAAAAAAAAACTCCCAGGGCCATTCCCTCCTTTGTGATACATACAGATTAGT

>*Alu*Yb9_element_hg19_Chr3:180,525,418_population_analysis

CTTATTGTAGAAAAATTTGAAAATAGAGATAAACCAAAAGAAAGATTATCCAATCCCCAAATTTTTGGCCAGAAAAATTACTGATTAGTGTATCTCTTTCCAGTGCTTCACATAAATATATATATACATATATAAACTAAACATATATGCAATAAAATACATGTCTATAAAGACAGTTACACATTTAAAATATATGTGTACATTTTAAATTTATATACACATATTTGTATATATACAGTTATAAAGTAAAATAGAGACAATTTTATATATTCTGTTTATATATTGAATATTAACTTGTTATGGACATCTTTATACCAAACAAATATGCTTCTATACCATATTTTTGTGATATTGTTGGACAACTTCAATTTAGATTGTTATAAAAAATACTGTGGGCCGGGCGCGGTGGCTCACGCCTGTAATCCCAGCACTTTGGGAGGCCGAGGCGGGTGGATCATGAGGTCAGGAGATCGAGACCATCCTGGCTAACAAGGTGAAACCCCGTCTCTACTAAAAATACAAAAAATTAGCCGGGCGCGGTGGCGGGCGCCTGTAGTCCCAGCTACTGGGGAGGCTGAGGCAGGAGAATGGCGTGAACCCGGGAAGCGGAGCTTGCAGTGAGCCGAGATTGCGCCACTGCAGTCCGCAGTCCGGCCTGGGCGACAGAGCGAGACTCCGTCTCAAAAAAAAAAAAAAAAAAAAACAAAAAAAAAACAAAAAAAAAACAAAAATACTGTGATGTGGTAAAGGGAACTTGAGGAGATACGGAAAAAAAGAAAAAATTAAAAAGGAAAAATACTGTGATGAACATACTTGTAGTTATAACCTTGCCTAAATTCTCAATTATCTCCTTAAGGCAAATTTCCAGGAGTAAGTTTTGATGCACATTGACAAATTGTCCTTGAGGAAGTTATATTTCCATCAGCAGTAGATAAAAGCTCTTGTTTTCATACATATTAACATAGAATATTTTCAGTTAATTTGTTGGCATTTATTAAAATCCCTATGTGCTCGTAGTTCTGTTCTAAATGGT

>*Alu*Yb8_element_hg19_Chr3:188,048,900_population_analysis

AACATGAAGCTGTCCAGCAGTAGAACAAATTGCCTTGCAAAGAGCTGCAAAAGTTCAAGTGGAGGCTACATAGCCATCTGTCAGAGAAGACATGTGATATCCTGGCAGTGGCTGGGAGTCTGCATTAAAAGATGGACATTGCAATTCTGGGTTTGGGAGATTTAGCAGCCCAATAGTGGGAGATACATAAAAGCTCAGATCTCCAGATAAAAATGTGAAGAACGCTCTCTAAAGAGGAGACCCCTGAGCAAAATCTATTCAACATATCCATGGGGCTCCTATGATACACCAAGCATGAGATAGCACCATCCTCAAGAAGCTGAGACGGCCGGGCGCGGTGGCTCACGCCTGTAATCCCAGCACTTTGGGAGGCCGAGGCGGGTGGATCATGAGGTCAGGAGATCGAGACCATCCTGGCTAACAAGGTGAAACCCCGTCTCTACTAAAAATACAAAAAATTAGCCGGGCGCGGTGGCGGGCGCCTGTAGTCCCAGCTACTCGGGAGGCTGAGGCAGGAGAATGGCGTGAACCCAGGAAGTGGAGCTTGCAGTGAGCCGAGATTGCGCCACTGCAGTCCGCAGTCTGGCCTGGGCGACAGAGCGAGACTCCGTCTCAAAAAAAAAAAAAAAAAAAAAAAAAAAAAGAAGCTGAGACAAGGAGGCAGAAAACAGGCATT

>*Alu*Yb8_element_hg19_Chr4:10,131,960_singleton_analysis

CAGAAGGGTGGGTGAACAGTCCACCCCCATGGAAGAGGTGAGTAGTTATCCGTTCACCCTAACATCCCTGAATGATTTCCCCATAACTGAAACAGAGCAGCTTTTCCTAGGGCCGAGTACACACCATCCAGACTCTGGCACCAGATTTCCAAAACACATCCAGAAACAGCTTGTCCTGTCGGTCAATCAGAAAGCAGCTTAATCGGCCGGGCGCGGTGGCTCACGCCTGTAATCCCAGCACTTTGGGAGGCCGAGGCGGGTGGATCATGAGGTCAGGAGATCGAGACCATCCTGGCTAACAAGGTGAAACCCCGTCTCTACTAAAAATACAAAAAATTAGCCGGGCGCGGTGGCGGGCGCCTGTAGTCCCAGCTACTCGGGAGGCTGAGGCAGGAGAATGGCGTGAACCCAGGAAGTGGAGCTTGCAGTGAGCCGAGATTGCGCCACTGCAGTCCGCAGTCTGGCCTGGGCGACAGAGCGAGACTCCGTCTCAAAAAAAAAAAAAAAAAAAAAAGAAAGCAGCTTAATCTAGTCTCAAAAGCCAGCACAGTTGTGCCGAGAAATGTCAGCATCTAAAATGCCATAAACAGCTAGGCTGTCTAAAATGAACTGTGATTATCAAGTTTGGCAGAGATTTGATCTTCACACGAAGTAGAGAGCTATGATTCAATAAACGAACTGAGAAGATCTAAGTAAAATAAGACTTCCTGAAGTTAAGGGTTCAAGTTCTTAAATACTCTTGGACTTGTGGTTTTAAATGCAGCAATTCAGTCATCTTTTCCACGTAGGATCTACGGTTTTACAGAAGTGAAGCTCTCACCAGTTCAAGTCTTATAATACCCGGACTGGTCTGACACTGGCCAGTTTCCTCATCTCTACAC

>*Alu*Yb8_element_hg19_Chr4:26,369,287_replication_analysis

CACTGCAGTAGGCACAGGAACTAATCCAAGAAAAATAAAATTTAAAATGTTACTGAGGCCGGGCGCGGTGGCTCACGCCTGTAATCCCAGCACTTTGGGAGGCCGAGGCGGGTGGATCATGAGGTCAGGAGATCGAGACCATCCTGGCTAACAAGGTGAAACCCCGTCTCTACTAAAAATACAAAAAATTAGCCGGGCGCGGTGGCGGGTGCCTGTAGTCCCAGCTACTCGGGAGGCTGAGGCAGGAGAATGGCGTGAACCCGGGAAGCGGAGCTTGCAGTGAGCCGAGATTGCGCCACTGCAGTCCGCAGTCCGGCCTGGGCGACAGAGCGAGACTCCGTCTCAAAAAAAAAAAAAAAAAAAAAAAAAAAAAAAAAAAAAAAAATGTTACTGAAATCTTCACAAAACAGA

>*Alu*Yb8_element_hg19_Chr4:59,817,993_singleton_analysis

GAAGGCTTCACACATATGCCCACCGAATTCTATAATTTCTGAGCTGTGCAAGTCCAAATAAAGTACAGCATATTGGTTAAATAAGCTTGTCATCCAAGTCAAATGTTCAGTTTTTATAAGTTTTCAACAATTGAAGTTATCATACCACTTCTTTCAATACTGAGATTTCAGGACTAATAAAGTTTAATCGGGATGTTCATACAGCTAAGTATTTATTTATAAAAATAAAATGTACCAACACCAGGAGCTGTGATAACAAGGTCAAATGACAACCTAGAATTGGATACTACAAGATGTTGCCATTCTAGTGAAATTAGGAGGAATAAAAACAACAAAAACTAAAACAACAAAAAGCAAAAACTAAACATCTACATATATAATTTCAATAAACAATTAAAAATAAATTACTCCTTTAATTGACTAGTGTAATTTATATAAGAAACACATATAGGGCCGGGCGCGGTGGCTCACGCCTGTAATCCCAGCACTTTGGGAGGCCGAGGCGGGTGGATCATGAGGTCAGGAGATCGAGACCATCCTGGCTAACAAGGTGAAACCCCGTCTCTACTAAAAATACAAAAAATTAGCCGGGCGCGGTGGCGGGCGCCTGTAGTCCCAGCTACTCGGGAGGCTGAGGCAGGAGAATGGCGTGAACCCGGGAAGCGGAGCTTGCAGTGAGCCGAGATTGCGCCACTGCAGTCCGCAGTCCGGCCTGGGCGACAGAGCGAGACTCCGTCTCAAAAAAAAAAAAAAAAAAAAAAAAAAAAAAAAAAAAAAAAAGAAACACATATAGGCTATCCCTGTAATAATATTAGTATTGAAAAATCAAAGAGTGATCCCTTTAATTAATTAATTAATTGTTTTGATGTTTTAATGATATTCATTGTTGTTCTTGAATACTCAAAAAGCCTCTGATATACTTCTCAATTATATCTTATTCTCTCAGGAGGTATCAGTAGAATCATACAGCCCTGTCTTC

>*Alu*Yb9_element_hg19_Chr4:60,532,747_singleton_analysis

TTGGGTCAAGGTACCACTGCTAGAAACCACTAGGGCAGGAATGGCTTAAGCCCCCATCAGATCTCTTTTATGAAAGTTAATGTAGCCGGGCGCGGTGGCTCACGCCTGTAATCCCAGCACTTTGGGAGGCCGAGGCGGGTGGATCATGAGGTCAGGAGATCGAGACCATCCTGGCTAACAAGGTGAAACCCCGTCTCTACTAAAAATACAAAAAATTAGCCGGGCGCGGTGGCGGGCGCCTGTAGTCCCAGCTACTGGGGAGGCTGAGGCAGGAGAATGGCGTGAATCCGGGAAGCGGAGCTTGCAGTGAGCCGAGATTGCGCCACTGCAGTCCGCAGTCCGGCCTGGGCGACAGAGCGAGACTCCGTCTCAAAAAAAAAAAAAAAAAAAAAAAAAAAAAAAAAAAAAAAAAAAAAAAAAAAAAAAAAAAAGAAAGTTAATGTATTGATTGGTGAAGAGAGACACTTTGATATTGTAACTGAGACACATTAGCCGAAATGAATAATCTGTATATCCAAAACTCCTACA

>*Alu*Yb8b1_element_hg19_Chr4:62,100,981_singleton_analysis

TGGCCTGGGAGTAATTACACATCTATGCTTTCTCCATCCTATGCCCATCTCATTGTTTACGGGATTTGTACTTTGCTTCTTTCTCACTTCTGTAAGCAACACTTTAGTCTACAAGCTCTAATACACTACAGTATCATTCTGAACTATAAACATTATAATTTGTAAAGCTTATAATCAAGGACATAGCCAGAAAAGATGTGTTAATTTAAAAACTGGCCAAATTATAAGTTTATACTACAAAAAAGAATTAGCAATCAACTAGTTCTCAACCCTTTCTTAATTATCACAAATACAGAGTAAAAACACAGGACAGGGCCGGGCGCGGTGGCTCACGCCTGTAATCCCAGCACTTTGGGAGGCCGAGGCGGGTGGATCATGAGGTCAGGAGATCGAGACCATCCTGGCTAACAAGGTGAAACCCCGTCTCTACTAAAAAATACAAAAAATTAGCCGGGCGCGGTGGCGGGCGCCTGTAGTCCCAGCTACTCGGGAGGCTGAGGCAGGAGAATGGCGTGAACCCGGGAAGCGGAGCTTGCAGTGAGCCGAGATTGCGCCACTGCAGTCCGCAGTCCGACCTGGGCGACAGAGCGAGACTCCGTCTCAAAAAAAAAAAAAAAAAAAAAAAAAAAAACACAGGACAGTTGAGTACTTTCATTTAATACATATCTACTGAGTACAAATTGTAGATAAGCAAGGCACATTTTTGCAGTGAGGATTGGGAGGCTGGAGGACTTAACATTTACTGAGTGTCATCTACTCATCGCTCTACTAGACAAACAAAGAGGTCGTTTCTCATTTATTTCTTATAGCCTTAAAGTAGATACTGTTATCTACATGTGCACAAGAGAAAAGAGGCTCATTTCCATCTCTTCTGGGACCTTATTTGGTTGGTTTTCTTGTTTTCCTCATGTTTTCACTTCTGGCTAACTTCATGCAACTCATAATCACATTCAAGCATCTCATATCTTGAAAGAGCAAATGAGCAAGGCAGTAAACAAAAACCAAACTTCACAGCTGTCTATGATTCTTATTTGCACACTGCCAAAACCTTCCTTTTTTTCTTTCCCAACCAATGGGGAAATAATGTACACCTGC

>*Alu*Yb9_element_hg19_Chr4:134,118,532_singleton_analysis

CCCCAATGGCCTTTCTGAATATGTATTTCCATACAAATAAATGCTTGTTGGAAATACTGCATATTTGACAGAATCATTTTGTTTGCATATGATTTTCATAAAAATGAATTTGGGCAGTATTCGGTATCTGCTCAATAGAATTGAATTTTAAAAGTTGCTCTAGGCCGGGCGCGGTGGCTCACGCCTGTAATCCCAGCACTTTGGGAGGCCGAGGCGGGTGGATCATGAGGTCAGGAGATCGAGACCATCCTGGCTAACAAGGTGAAACCCCGTCTCTACTAAAAAATACAAAAAATTAGCCGGGCGCGGTGGCGGGCGCCTGTAGTCCCAGCTACTGGGGAGGCTGAGGCAGGAGAATGGCGTGAACCCGGGAAGCGGAGCTTGCAGTGAGCCGAGATTGTGCCACTGCAGTCCGCAGTCCGGCCTGGGCGACAGAGCGAGACTCCGTCTCAAAAAAAAAAAAAAAAAAAAAAAAAAAGTTGCTCTAATAGTAGACTTCTTGAAGTTG

>*Alu*Yb8_element_hg19_Chr4:149,524,501_population-panel_analysis

CGTATGTTCAGTGCTCAGTTTTAATCTGTCAAACAGTAGTTATGGTTGTTAAAACAATTTATACAATCAATAGTGGTGTATTAAAAATTATCCTGGATTGGCCGGGCGCGGTGGCTCACGCCTGTAATCCCAGCACTTTGGGAGGCCGAGGCGGGTGGATCATGAGGTCAGGAGATCGAGACCATCCTGGCTAACAAGGTGAAACCCCGTCTCTACTAAAAATACAAAAAATTAGCCGGGCGCGGTGGCGGGCGCCTGTAGTCCCAGCTACTCGGGAGGCTGAGGCAGGAGAATGGCGTGAACCCGGGAAGCGGAGCTTGCAGTGAGCCGAGATTGCGCCACTGCAGTCCGCAGTCCGGCCTGGGCGACAGAGCGAGACTCCGTCTCAAAAAAAAAAAAAAAAAAAAAAAAAAAAAAATTATCCTGGATTATTTTTATTGTTGTTTGCCATTTTTATTTGACTATATTTGGAGAATATATTCTGCAGAACCTGTACTTCTTGAAAGGTAAAATTTTAATTGTGACCAAGAACATGAACAACTTTTAAAAATATTCCCTGAACTAACAGAAAAAATGTATAGTCTATGCTTTTTTATATACACACACAATCATCAAAT

>*Alu*Yb8_element_hg19_Chr4:170,604,831_population_analysis

ATTAGGTTGGGTATATGCACTGACATAGAAAGGCTTTAAAATTTAATGTAGGCCGGGCGCAGTGGCTCCCACCTGTAATCCCAACATTTTGGGAGGCTGAGACAGGTGGATCACTTGAGGCCGGGAGTTTGAGACCAGCCTGGCCAACATGGCGAAACCACATCTCTACTTAAACTACAAAAATTAGCCAGTGTGGTGGCGTATGCCTGTAATCCCAGTTACTTGGGGTGCTGAAGCACGAGAGCTGCTTGAGCCTGGAAGGCAGAGGTTGCAGGGAGTCGAGATCGCACCACTGAACTTCAGCCTGGGCAAAAGAGCAAGACTCTGCTTAAAAAAAAAAAAAAAAAATTAATTTTAAGAACAATAGTAAGTATATAAATGTGTGGAATTTTCCAGCTTTAAAAAATCTTGTTGGCCGGGCGCGGTGGCTCACGCCTGTAATCCCAGCACTTTGGGAGGCCGAGGCGGGTGGATCATGAGGTCAGGAGATCGAGACCATCCTGGCTAACAAGGTGAAACCCCGTCTCTACTAAAAATACAAAAAATTAGCCGGGCGCGGTGGCGGGCGCCTGTAGTCCCAGCTACTCGGGAGGCTGAGGCAGGAGAATGGCGTGAACCCGGGAAGCGGAGCTTGCAGTGAGCCGAGATTGCGCCACTGCAGTCCGCAGTCCGGCCTGGGCGACAGAGCGAGACTCCGTCTCAAAAAAAAAAAAAAAAAAAAAAAAAAAAAAAAAAAAAAAAAAAAAAAAAAAAAAAAAAAAAAAAAAAATCTTGTTCATATAAGCATGGATCAAAATATT

>*Alu*Yb8b1_element_hg19_Chr5:23,723,353_singleton_analysis

TTCTGACTTCTCTAAGCAATCACCTTATACATTTAATAACTTAGTATTTTCAATTGCATAAGTCAAAACTACCTTAAAGTAGACAAACTGTGACAAAGCAGAACTCATGTACTCCTTCATAAAAATTATCCTCAGTGGGCCGGGCGCGGTGGCTCACGCCTGTAATCCCAGCACTTTGGGAGGCCGAGGCGGGTGGATCATGAGGTCAGGAGATCGAGACCATCCTGGCTAACAAGGTGAAACCCCGTCTCTACTAAAAATACAAAAAATTAGCCGGGCGCGGTGGCGGGCGCCTGTAGTCCCAGCTACTCGGGAGGCTGAGGCAGGAGAATGGCGTGAACCCGGGAAGCGGAGCTTGCAGTGAGCCGAGATTGCGCCACTGCAGTCCGCAGTCCGACCTGGGCGACAGAGCGAGACTCCGTCTCAAAAAAAAAAAAAAAAAAAAAAATTATCCTCAGTGATAAAATAAATTAAATCTACTCATTCCAAATAAGTCAGAATCATTCTTAATATTTCCTTTTTTAATACCAGAATATATCTCACCAATTGCTTTCACTTTTACCCATATCTTCTCTGTGTCTTTCTTCACAACCATCTACCTAGACAAAGTTACCATAAACTCTTGCCTGACTTCTGCAATAGTTTTGTATTATCTCTCCCCAGTCGTCTTTCTTTAAATAGTTTATA

>*Alu*Yb8_element_hg19_Chr5:31,211,126_singleton_analysis

ATAACAAGACCCAAATCCACGAATTCTGTTCAATGAGGGTTACCTGCAAAATGACAATTTAGGGCCGGGCGCGGTGGCTCACGCCTGTAATCCCAGCACTTTGGGAGGCCGAGGCGGGTGGATCATGAGGTCAGGAGATCGAGGCCATCCTGGCTAACAAGGTGAAACCCCGTCTCTACTAAAAATACAAAAAATTAGCCGGGCGCGGTGGCGGGCGCCTGTAGTCCCAGCTACTCGGGAGGCTGAGGCAGGAGAATGGCGTGAACCCGGGAAGCGGAGCTTGCAGTGAGCCGAGATTGCGCCACTGCAGTCCGCAGTCCGGCCTGGGCGACAGAGCGAGACTCCGTCTCAAAAAAAAAAAAAAAAAAAAAAAAAAAAAAAAAAAAAAAAAAAAAAAAAAAAAAAAAAAAAAAAAAAAAAAAAATGACAATTTAGAGCAATACTAATTATTGAAGGACCT

>*Alu*Yb7.2_element_hg19_Chr5:148,143,449_doubleton_analysis

TCTCAGGGGATTTCAACTGTAAGAAAAAGTACAAAAAAATCTCCAACCACATTGAAAAGTTTGAAGGTATAAGAATGAGGGCCGGGCGCGGTGGCTCACGCCTGTAATCCCAGCACTTTGGGAGGCCGAGGCGGGTGGATCATGAGGTCAGGAGATCGAGACCATCCTGGCTAACAAGGTGAAACCCCGTCTCTACTAAAAATACAAAAAATTAGCCGGGCGTAGTGGCGGGCGCCTGTAGTCCCAGCTACTCGGGAGGCTGAGGCAGGAGAATGGCGTGAACCCGGGAAGCGGAGCTTGCAGTGAGCCGAGATTGCGCCACTGCAGTCCGCAGTCCGGCCTGGGCGACAGAGCGAGACTCCGTCTCAAAAAAAAAAAAAAAAAAAAAAAAAAAAAAAAAGAATGAGAGTAGGAGATCCTTTTGAAAATCAAAATTCTCTATTTTTTGAAATAATATTTAAATATTAATGTTTGTGGACCAATATAGTAACCCAAGAGACTCTTCTTTTGAAGACAAATTCTAAT

>*Alu*Yb8b1_element_hg19_Chr5:168,478,425_population-panel_analysis

>Sanger sequencing of forward primer failed (too close to poly(A) tail)

CTCTGTGATCAGTGAGTTTAAATCAGAATAGGTCTTAGGTAGCCAAAGTGGCTTAAAATGTTAGCAAACAGTGGGATCCCTTTTAATTTTTAAAGGGAACTCTAATTCTACCCTTTATGACTTGGTTAATTATCCTGGGCCCCTGAACAGCTGTCTATTGATTCCAATTAACATGATTGACATCTCCTCTGGAAGGTAAAAGCTAATCAACTACAGCATCTCCCACTGAGAATTCGCGCGCGGTGGCTCACGCCTGTAATCCCAGCACTTTGGGAGGCCGAGGCGGGTGGATCATGAGGTCAGGAGATCGAGACCATCCTGGCTAACAAGGTGAAACCCCGTCTCTACTAAAAATACAAAAAATTAGCCGGGCGCGGTGGCGGGCGCCTGTAGTCCCAGCTACTCGGGAGGCTGAGGCAGGAGAATGGCGTGAACCCGGGAAGCGGAGCTTGCAGTGAGCCGAGATTGCGCCACTACAGTCCGCAGTCCGACCTGGGCGACAGAGCGAGACTCCGTCTCAAAAAAAAAAAAAAAAAAAAAAAAAAAAAAAAAAAAAAAAAAAAAAAAAA

>*Alu*Yb8b1_element_hg19_Chr6:110,423,938_replicate_analysis

CAGAGAGGATTGGCAGTTGCTTATAATAATCTTTCAGTAAAGCTATTTTGAAGTTTAATCTTGTCAGTTATTATCCAAATGATAAATTTCTAATAGAGAAATATTAGGGAAATTTTCTAATAGAGATATAGAGATCAGCTTTACAATTTTCAGTTAAAAATCCAATTTTCGGCCGGGCGCGGTGGCTCACGCCTGTAATCCCAGCACTTTGGGAGGCCGAGGCGGGTGGATCATGAGGTCAGGAGATCGAGACCATCCTGGCTAACAAGGTGAAACCCCGTCTCTACTAAAAATACAAAAAATTAGCCGGGCGCGGTGTCGGGCGCCTGTAGTCCCAGCTACTCGGGAGGCTGAGACAGGAGAATGGCGTGAACCCGGGAAGCGGAGCTTGCAGTGAGCCGAGATTGCGCCACTGCAGTCCGCAGTCCGACCTGGGCGACAGAGCGAGACTCCGTCTCAAAAAAAAAAAAAAAAAAAAAAAAAAAAATCCAATTTTCTATCACTGACTCATTTGGTGTTCCATCCTCAGGAAAAGAGAGAGCTGTAAATTTCTTTTTGTGGTTTGTAATTAGCTGTCATTGATTTAACTGAAATAGCATATGATGAGACTTCATAG

>*Alu*Yb8b1_element_hg19_Chr6:151,163,476_UTR_analysis

GTATTCTCATTTTAATCAGGTGTACATTCTATGGCCTCTCCCCCATGCTGTTAGTTTCTATTTTAAAAGATACAATAGGCCGGGCGCGGTGGCTCACGCCTGTAATCCCAGCACTTTGGGAGGCCGAGGCGGGTGGATCATGAGGTCAGGAGATCGAGACCATCCTGGCTAACAAGGTGAAACCCCGTCTCTACTAAAAATACAAAAAATTAGCCGGGCGCGGTGGCGGGCGCCTGTAGTCCCAGCTACTCGGGAGGCTGAGGCAGGAGAATGGCGTGAACCCGGGAAGCGGAGCTTGCAGTGAGCCGAGATTGCGCCACTGCAGTCCGCAGTCCGACCTGGGCGACAGAGCGAGACTCCGTCTCAAAAAAAAAAAAAAAAAAAAAAAAGATACAATAATATATGTAGGGAAAGGGGCCTGGGCTCTTCATTTAAAGGTAAGCAGTAATATTGAGTAAGTGACATAATTCTTTTTCTCTTTGTTAAGTCCTATGCCTCTTTTTCTTAACTGTAAAACATAGAATATGAG

>*Alu*Yb8b1_element_hg19_Chr7:20,613,951_singleton_analysis

TGAGAGAACCGTCTCCCATACTAAGGACATCTTTGTTTAGATAGATTTAATGTTTCTAACTATACTTTTTAAAGTATAATCAAATTCATAAATTGTACACCGCAAATACTCTTTGTAAAGGCCCAAGTTTTTCTGCCTTTATTTATCTTGCAAATATTCATCTACACTGAATGTTCTTTTAAAAGTTACATATTACGTGGCTCACGCCTGTAATCCCAGCACTTTGGGAGGCCGAGGCGGGTGGATCATGAGGTCAGGAGATCGAGACCATCCTGGCTAACAAGGTGAAACCCCGTCTCTACTAAAAAAAAAATACAAAAAATTAGCCGGGCGCGGTGGCGGGCGCCTGTAGTCCCAGCTACTCGGGAGGCTGAGGCAGGAGAATGGCGTGAACCCGGGAAGCGGAGCTTGCAGTGAGCCGAGATTGCGCCACTGCAGTCCGCAGTCCGACCTGGGCGACAGAGCGAGACTCCGTCTCAAAAAAAAAAAAAAAAAAAAAAAAAAAAAAAAAAAAGTTACATATTACATATTTTTATTTGTAGATCAAAGTGCTGAATAAACAATTATCTCTCTGATACACCAATGACATCCTTTAAATATAGCTCACTACATTAAAAACCATTCAAGTTCAATGAGAATATCAGCTATCAAATTGAATATCAATAATAGTTATATTCAGATGAGGGGATCAGAATGTTAAATTTCAAATGGATTACTAGACTATCAAACTCTTCTCCATCATCCTGGAGATACTGGCACATAATACACTTCTTCTTCTCTGAAAAGTGCAGTAAACAAAGTATAGTCATGGTAGGAATTTAAAATGATACATCAAGGAAATATTGATGTTGACCCAAATGTTGCTCTCTTTTTTAAACTTTCTTGAAGACCCAGAAGAACCAATATTTTATGACTCCCAGCTTAGATTGGCCCCCCACGTCT

>*Alu*Yb11_element_hg19_Chr7:36,932,555_doubleton_analysis

AGTGGGCCATGGTACTTCTTGGTCCATGGCCGAGGGATTATGCAAAAGCTACATCCATGCAGGAGATTTCCTTCATATTTGGATAGTTTCAAAGATAAGAGCATCTTGGCCGGGCGCGGTGGCTCACGCCTGTAATCCCAGCACTTTGGGAGGCCGAGGCGGGTGGATCATGAGGTCAGGAGATCGAGACCATCCTGGCTAACAAGGTGAAACCCCGTCTCTACTAAAAATACAAAAAATTAGCCGGGCGCGGTGGCGGGCGCCTGTAGTCCCAGCTACTGGGGAGGCTGAGGCAGGAGAATGGCGTTGAACCCGGGAAGCGGAGCTTGCAGTGAGCCGAGATTGCGCCACTGCAGTCCGCAGTCCAGCCTGGGCGACAGAGCGAGACTCCGTCTCAAAAAAAAAAAAAAAAAAAAAAAAAAAAAGATAAGAGCATCTTATAATAAAGGGGAAAGAAACTTCAGAATGTCACAGGCAAAGTCTTAGGAAGTGAAGATATAAAGCCAAAATAGGAGTAAAATGTTCGTACTTGCCATGAAGTTAATTAACATTCTTACTTGGAAAAAGTCAAGGTCAAACATAATTCATCAAACATAATTCAGAGTTACTTGGCTTTAACGGAAGTGATTCCTCAAGTCTCCCTTCAGGTTTTAAGACTTATTATGGAAAGCATAATAAAGTGTCCTACGTTTATACATCTGCTGAACCATGTTATGAAATCCAGTACACTGTAGGCCCTGGGATGGGGCAAGGAGCCATTGTCCCTCTTTGCAGGTGAGAAACTGCATCTCTGAGGAGCCAGTCACTTATCCAAAGTGCATCCTGCTTGG

>*Alu*Yb9_element_hg19_Chr7:140,176,373_population_analysis

GTAGTTCCAGAAAAAATATACATGTGGCCGGGCGTAATCCCACCTACTCAGGAGGCTGAGGCAGGAGAATCGCTTGAACCCAGGAGGCAGAGGTTGCAGTGAGCTGAGATCTCGCCACTGCACTCCAGCCTGGGCGACAGAGCGACACTCCATCTCAAAAAAAAAAAAAAAAAAAAGGAAGATACACATGTGTTCTGTTAAGTACTTTGTCTTTGGTAATTTACAAGTTTTGTTTTTTTTTTTTTTAATGAAAGCAGAGGGGCCGGGCGCGGTGGCTCACGCCTGTAATCCCAGCACTTTGGGAGGCCGAGGCGGGTGGATCATGAGGTCAGGAGATCGAGACCATCCTGGCTAACAAGGTGAAACCCCGTCTCTACTAAAAATACAAAAAATTAGCCGGGCGCGGTGGCGGGCGCCTGTAGTCCCAGCTACTGGGGAGGCTGAGGCAGGAGAATGGCGTGAACCCGGGAAGCGGAGCTTGCAGTGAGCCGAGATTGCGCCACTGCAGTCCGCAGTCCGGCCTGGGCGACAGAGCGAGACTCCGTCTCAAAAAAAAAAAAAAAAAAAAAAAAAAAAAAAAAAAAAAAAAAAAAAAAAAAAAGAAAGCAGAGGAATCTTACAAAGTTCCTGTTAAAGTGGGTAATTCTAGGTATATGACTTTATCAACATCACTCAAGCATTTACTCAGTATCTGCTCAGGATACTCAGTAACTGAG

>*Alu*Yb8b1_element_hg19_Chr8:16,524,642_replicate_analysis

CCTTGGGAGAACAAAATACACTCTTTGTGTTGCTGTTGGCTGGTACAGAAAGTATTGGCATGAGTTAATCTACCATTGTTGTGGGTAGAAAAAAATACTCTTTTTCTCTGCTTGAATCCTAAGACTAGTTACAAGTTTTCACAACAAATAAGATGATATAAGAATAGTACTTGGCCGGGCGCGGTGGCTCACGCCTGTAATCCCAGCACTTTGGGAGGCCGAGGCGGGTGGATCATGAGGTCAGGAGATCGAGACCACCCTGGCTAACAAGGTGAAACCCCGTCTCTACTAAAAATACAAAAAATTAGCCGGGCGCGGTGGCGGGCGCCTGTAGTCCCAGCTACTCGGGAGGCTGAGGCAGGAGAATGGCGTGAACCCGGGAAGCGGAGCTTGCAGTGAGCCGAGATTGCGCCACTGCAGTCCGCAGTCCGACCTGGGCGACAGAGCGAGACTCCGTCTCAAAAAAAAAAAAAAAAAAAAAAAAAAAAAAAAAAAAAAAAAAAAAAAAAAAAAAAAAAGAATAGTACTTGGCTTTTTATACAGTAAATTACACCAGTGACATGTAAAAGGTGTAGAGAAAAAAATATTCTCTGACAC

>*Alu*Yb7.1_element_hg19_Chr8:23,013,832_population_analysis

>Reverse primer too close to poly(A) tail, could not distinguish second TSD.

AAGATAAAACAGCAATTCAGGGATTATGGCCATGATCTCTGTGTTATAAATGACGACGAGAAGACAACTGGAGAATCGGAATAGAGTCTGCATATTAGATAGTGGTAATGTGTTGATGTTATTTTCCTTGTTTGTAGAAAGTACACACAGAAAAATTCAATAATAGCTTTCATCATGCCAACAGCTATTTTTCAAATTTTTTTGCATAGTTTTTGTAACTTTTAAGTTGGGAGTGTGTAAAAGTACAAAATTAATTTAATTGTAAATTTACATTTAATTAAATTTAATATAATCTTGCTCCTGAGAAAAAACAAAATGGTTACAATAAAAGCTACTTACCGCCTGTAATCCCAGCACTTTGGGAGGCCGAGGCGGGTGGATCATGAGGTCAGGAGATCGAGACCATCCTGGCTAACAAGGTGAAACCCCGTCTCTACTAAAAATACAAAAAATTAGCCGGGCGCGGTGGCGGGCGCCTGTAGTCCCAGCTACTCGGGAGGCTGAGGCAGGAGAATGGCGTGAACCCGGGAAGCGGAGCTTGCAGTGAGCCGAGATCGCGCCACTGCAGTCCGCAGTCCGGCCTGGGCGACAGAGCGAGACTCCGTCTCAAAAAAAAAAAAAAAAAAAAAAAAAAAA

>*Alu*Yb8_element_hg19_Chr8:84,702,094_population-panel_analysis

TCCTTACACATTACTGGGATGGCTAATTTTTATGTATCAAATTGAGCTAAGTGCTGTCAAGATAGTTGGTAAAACATTATTTCTGGCCGGGCGCGGTGGCTCACGCCTGTAATCCCAGCACTTTGGGAGGCCGAGGCGGGTGGATCATGAGGTCAGGAGATCGAGACCATCCTGGCTAACAAGGTGAAACCCCGTCTCTACTAAAAATACAAAAAATTAGCCGGGCGCGGTGGCGGGCGCCTGTAGTCCCAGCTACTCGGGAGGCTGAGGCAGGAGAATGGCGTGAACCCGGGAAGCGGAGCTTGCAGTGAGCCGAGATTGCGCCACTGCAGTCCGCAGTCCGGCCTGGGCGACAGAGCGAGACTCCGTCTCAAAAAAAAAAAAAAAAAAAAAAAAAAAAAAAAAAAAAAAAAAAAAAAAAAAAAAACATTATTTCTGAGTGTGTCTTTGAGGGTGTTTCTGGACAATATTAGCATTTGAATCAGTCTACTAAGTACAGAGGTCCA

>*Alu*Yb8b1_element_hg19_Chr8:109,813,022_population-panel_analysis

AAATGTTCCTTCTCCAAGCTAGCTTATACTACATTGGGAAAAAACTTGAAAGTGGATAAATTAATCATATATTCCTCTGTCTTTGAATTCCCAGCATCTAACATAGTGCCTGACCAGAGTATGTGCTGAATAAAATTTACTAAATGTGTGAAGCAATAAATGTGCAGACTGCAACATCATAAAAAGTCACCAACTAGGCCGGGCGCGGTGGCTCACGCCTGTAATCCCAGCACTTTGGGAGGCCGAGGCGGGTGGATCATGAGGTCAGGAGATCGAGACCATCCTGGCTAACAAGGTGAAACCCCGTCTCTACTAAAAATACAAAAAATTAGCCGGGCGCGGTGGCGGGCGCCTGTAGTCCCAGCTACTCGGGAGGCTGAGGCAGGAGAATGGCGTGAACCCGGGAAGCGGAGCTTGCAGTGAGCCGAGATTGCGCCACTGCAGTCCGCAGTCCGACCTGGGCGACAGAGCGAGACTCCGTCTCAAAAAAAAAAAAAAAAAAAAAAAAAAAAAAAAAAAAAAAAAAAAAAAAAAAAAAAAAAAAAAGTCACCAACTATAACTAGCAAAG

>*Alu*Yb8_element_hg19_Chr8:115,678,706_replicate_analysis

>Reverse primer too close to poly(A) tail, could not distinguish TSD.

TTTCCCTTCGTTGAATCATTTTTAATGAAATTCTAGGAAATATTCAGGATAAAATTCTAGTCATATAATTTAGTCAATATAAACAGGAATCGGCCGGGCGCGGTGGCTCACGCCTGTAATCCCAGCACTTTGGGAGGCCGAGGCGGGTGGATCATGAGGTCAGGAGATCGAGACCATCCTGGCTAACAAGGTGAAACCCCGTCTCTACTAAAAATACAAAAAATTAGCCGGGCGCGGTGGCGGGCGCCTGTAGTCCCAGCTACTCGGGAGGCTGAGGCAGGAGAATGGCGTGAACCCGGGAAGCGGAGCTTGCAGTGAGCCGAGATTGCGCCACTGCAGTCCGCAGTCCGGCCTGGGCGACAGAGCGAGACTCCGTCTCAAAAAAAAAAAAAAAAAAAAAAAAAA

>*Alu*Yb7_element_hg19_Chr8:116,728,191_population_analysis

TGAAGGCAGATCAGGTTGGCTCATTAATTTTTGCCGTGAGAAGACAAAGGAGATAATGTAATCGGGTTTTTAGTATATTATATTTTTCAACAATTATGCAGAACTTTCACACTTTCTGATTTTATGGAAACTTTTTATTTAAAAATTGTTAGGCCGGGCGCGGTGGCTCACGCCTGTAATCCCAGCACTTTGGGAGGCCGAGGCGGGTGGATCATGAGGTCAGGAGATCGAGACCATCCTGGCTAACAAGGTGAAACCCCGTCTCTACTAAAAATACAAAAAATTAGCCGGGCGCGGTGGCGGGCGCCTGTAGTCCCAGCTACTCGGGAGGCTGAGGCAGGAGAATGGCGTGAACCCGGGAAGCGGAGCTTGCAGTGAGCCGAGATTGCGCCACTGCAATCCGCAGTCCGGCCTGGGCGACAGAGCGAGACTCCGTCTCAAAAAAAAAAAAAAAAAAAAAAAAAAAAAAAAAAAAAAAAAAAAAAAAAAAAAAAAAAAAAAAAAAAAAAAAAAAAAAAAAAAAAAAAAAAAAAAAAAAAAAAAAAAAAAATTGTTAAACCTGGCTCATTTGAGGTCTAAATATATGTAAGGCAAATAGAACTCAAGCCAAATTTATGTCTTTGGTAGCAAAAGTTCTACATTTGGAAGCATATTCTATAATAGGTAAATATCAGAATAGAAAGGTTGGGGTTTGGAGAGTTTTACTTAAAACTATCACTTTAAATTTTGTATTTTTCATTTATATTT

>*Alu*Yb9_element_hg19_Chr9:106,211,943_replicate_analysis

GCACTCAAAAATGGTTAATACGGTACATTTCAGATCATGTGTATTTAATATAATTTTAAAAATAAAATACAGGCCGGGCGCGGTGGCTCACGCCTGTAATCCCAGCACTTTGGGAGGCCGAGGCGGGTGGATCATGAGGTCAGGAGATCGAGACCATCCTGGCTAACAAGGTGAAACCCCGTCTCTACTAAAAATACAAAAAATTAGCCGGGCGCGGTGGCGGGAGCCTGTAGTCCCAGCTACTGGGGAGGCTGAGGCAGGAGAATGGCGTGAACCCGGGAAGCGGAGCTTGCAGTGAGCCGAGATTGCGCCACTGCAGTCCGCAGTCCGGCCTGGGCGACAGAGCGAGACTCCGTCTCAAAAAAAAAAAAAAATAAAATAAAATACAATAGAAAACACATAAGAAAAGCTAATAATAGATATCATTCCGTAGTGAT

>*Alu*Yb8_element_hg19_Chr9:114,889,844_population-panel_analysis

ATTGGCTCTCCCTAAGCTCCATGAGGGCAGAAATCTTACCCACTTTGTTCACTGCTATAATGACTAGCATGTAATAGGCACTCAATAGACAGGAATAAATAAACGAATGAACATAGATCTTAAAAGTTCAGGCCGGGCGCGGTGGCTCACGCCTGTAATCCCAGCACTTTGGGAGGCCGAGGCGGGTGGATCATGAGGTCAGGAGATCGAGACCATCCTGGCTAACAAGGTGAAACCCCGTCTCTACTAAAAATACAAAAAATTAGCCGGGCGCGGTGGCAGGCGCCTGTAGTCCCAGCTACTCGGGAGGCTGAGGCAGGAGAATGGCGTGAACCCGGGAAGCGGAGCTTGCAGTGAGCCGAGATTGCGCCACTGCAGTCCGCAGTCCGGCCTGGGCGACAGAGCGAGACTCCGTCTCAAAAAAAAAAAAAAAAAAAAAAGTTCAGAGGAAGACAGGATCCCTTTGTTAGTAATGGAGCAGATCAGGCACGGGGAAGGACTTGCTCAGTG

>*Alu*Yb7.2_element_hg19_Chr9:114,940,676_population-panel_analysis

TTTGGAGGAACAGTGGGATTATTGCTGACAAAGTATTTGACTTAAGAAAAATGTTCTTTCAAGAGAGCATGGTTGCCTGTAATCCCAGCACTTTGGGAGGCCGAGGCGGGTGGATCATGAGGTCAGGAGATCGAGACCATCCTGGCTAACAAGGTGAAACCCCGTCTCTACTAAAAATTCAAAAAATTAGCCGGGCGTGGTGGCGGGCGCCTGTAGTCCCAGCTACTCGGGAGGCTGAGGCAGGAGAATGGCGTGAACCCGGGAAGCGGAGCTTGCAGTGAGCCGAGATTGCGCCACTGCAGTCCGCAGTCCGGCCTGGGCGACAGAGCGAGACTCCGTCTCAAAAAAAAAAAAAAAAAAAAAAAAAAAAAAGAGAGCATGGTTAATATACCAGCATTTTCTAAAAAAGAACTTGAAAGAAGCTTTCTTCTCCTTCTCCCTCTCCTCCATTTCCTCCTCATTCTTCTTCTTCTT

>*Alu*Yb8b1_element_hg19_Chr9:122,357,545_replicate_analysis

GGCATAAAACGTGGGATCATATTGTTGAAATATTTAAGAGGGCCGGGCGCGGTGGCTCACGCCTGTAATCCCAGCACTTTGGGAGGCCGAGGCGGGTGGATCATGAGGTCGGGAGATCGAGACCATCCTGGCTAACAGGGTGAAACCCCGTCTCTACTAAAAAAAAAATACAAAAAATTAGCCGGGCGCGGTGGCGGGCGCCTGTAGTCCCAGCTACTCGGGAGGCTGAGGCAGGAGAATGGCGTGAACCTGGGAAGCGGAGCTTGCAGTGAGCCGAGATTGCGCCACTGCAGTCCGCAGTCCGACCTGGGCGACAGAGCGAGACTCCGTCTCAAAAAAAAAAAAAAAAAAAAAAAAAAAAAGAAATATTTAAGAAATACTAGAATAAAGCA

>*Alu*Yb8_element_hg19_Chr10:20,194,540_population_analysis

CAACGACACCAGGAAAAAAGTCTAATATTTTGTAACTTCAGGGTACCTTTTGCAATTAAAGAATTCATTCTAGGGTATTCAAAAGGAGCTAATTATTCTAAATCATAATATAATTGGGAGTGCAATTGAGAATTTTCTATGGCTGACTGGTTGAATTAGAAAAGCAAATCCAGGCCGGGCGCGGTGGCTCACGCCTGTAATCCCAGCACTTTGGGAGGCCGAGGCGGGTGGATCATGAGGTCAGGAGATCGAGACCATCCTGGCTAACAAGGTGAAACCCCGTCTCTACTAAAAATACAAAAAATTAGCCGGGCGCGGTGGCGGGCGCCTGTAGTCCCAGCTACTCGGGAGGCTGAGGCAGGAGAATGGCGTGAACCCGGGAAGCGGAGCTTGCAGCGAGCCGAGATTGCGCCACTGCAGTCCGCAGTCCGGCCTGGGCGACAGAGCGAGACTCCGTCTCAAAAAAAAAAAAAAAAAAAAAAAAAAAGAAAAGCAAATCCAAAAAGCACTCAGTAT

>*Alu*Yb8b1_element_hg19_Chr10:66,695,823_replicate_analysis

TTCATTTCCTCAAAATCATGCACTTATTTATCTTTAATGATGTAAACATAAGAATGTATAATGTTGGCCGGGCGCGGTGGCTCACGCCTGTAATCCCAGCACTTTGGGAGGCCGAGGCGGGTGGATCATGAGGTCAGGAGATCGAGACCATCCTGGCTAACAAGGTGAAACCCCGTCTCTACTAAAAATACAAAAAATTAGCCGGGCGCGGTGGCGGGCGCCTGTAGTCCCAGCTACTCGGGAGGCTGAGGCAGGAGAATGGCGTGAACCCGGGAAGCGGAGCTTGCAGTGAGCCGAGATTGCGCCACTGCAGTCCGCAGTCCGACCTGGGCGACAGAGCGAGACTCCGTCTCAAAAAAAAAAAAAAAAAAAAAAAAAAAGAATGTATAATGTTATTGCTGCAAATGAATCTGTCTTAATTCTCAAAAACAAGAAAACTAATAAATGTTAAATACCTCATA

>*Alu*Yb8_element_hg19_Chr10:87,351,114_replicate_analysis

GGTAAACCATGCTATGGAAGAAAAGAGACAGCTCGGGCGCGGTGGCTCACGCCTGTAATCCCAGCACTTTGGGAGGCCGAGGCGGGTGGATCATGAGGTCAGGAGATCGAGACCATCCTGGCTAACAAGGGGAAACCCCGTCTCTACTAAAAATACAAAAAATTAGCCGGGCGCGGTGGCGGGCGCCTGTAGTCCCAGCTACTCGGGAGGCTGAGGCAGGAGAATGGCGTGAACCCGGGAAGCGGAGCTTGCAGTGAGCCGAGATTGCGCCACTGCAGTCCGCAGTCTGGCCTGGGCGACAGAGCGAGACTCCGTCTCAAAAAAAAAAAAAAAAAAAAAAAAAAAAAAAAAAAGAGACAGCTCACAAAAGCAATATACAAATCGGCATATGTAAAATGCGCAATCTCATTAATCATCAGTAAA

>*Alu*Yb8_element_hg19_Chr10:117,017,772_replicate_analysis

AAGCGAGAAGAAAAAGTAAGAAAAAACAGACTAAAAAGAAATGAACAGGCCGGGCGCGGTGGCTCACGCCTGTAATCCCAGCACTTTGGGAGGCCGAGGCGGGTGGATCATGAGGTCAGGAGATCGAGACCATCCTGGCTAACAAGGTGAAACCCCGTCTCTACTAAAAATACAAAAAATTAGCCGGGCGCGGTGGCAGGCGCCTGTAGTCCCAGCTACTCGGGAGGCTGAGGCAGGAGAATGGCGTGAACCCGGGAAGCGGAGCTTGCAGTGAGCCGAGATTGCGCCACTGCAGTCCGCAGTCCGGCCTGGGCGACAGAGCGAGACTCCGTCTCAAAAAAAAAAAAAAAAAAAAAAAAAAAAAAAAAAAAAAAAAAAAGAAATGAACAAAGCCTCCAAGAAATATGGGACTATGTGAAAAGACCAA

>*Alu*Yb8b1_element_hg19_Chr11:10,470,454_replicate_analysis

TCAGCTGTTCTTGCAAATCATTTTAACAAATGGGACTTTTAAAACACTTGAGGCCGGGCGCGGTGGCTCACGCCTGTAATCCCAGCACTTTGGGAGGCCGAGGCGGGTGGATCATGAGGTCAGGAGATCGAGACCATCCTGGCTAACAAGGTGAAACCCCGTCTCTACTAAAAATACAAAAAATTAGCCGGGCGCGGTGGCGGGCGCCTGTAGTCCCAGCTACTCGGGAGGCTGAGGCAGGAGAATGGCGTGAACCCGGGAAGCGGAGCTTGCAGTGAGCCGAGATTGCGCCCACTGCAGTCCGCAGTCCGGCCTGGGCGACAGAGCGAGACTCCGTCTCAAAAAAAAAAAAAAAAAAAAAAAAAAAAAAAAAAAAACACTTGAAATTCTGCATTGCAATAT

>*Alu*Yb11_element_hg19_Chr11:82,102,374_replicate_analysis

TTTCCGCCACTTCCTTAATCTTTGTGAGGCCTTCTGAATAATTTTTTAAAAATGATTTTCAGGCCGGGCGCGGTGGCTCACGCCTGTAATCCCAGCACTTTGGGAGGCCGAGGCGGGTGGATCATGAGGTCAGGAGATCGAGACCATCCTGGCTAACAAGGTGAAACCCCGTCTCTACTAAAAATACAAAAAATTAGCCGGGCGCGGTGGCGGGCGCCTGTAGTCCCAGCTACTGGGGAGGCTGAGGCAGGAGAATGGCGTTGAACCCGGGAAGCGGAGCTTGCAGTGAGCCGAGATTGCGCCACTGCAGTCCGCAGTCCGGCCTGGGCGACAGAGCGAGACTCCGTCTCAAAAAAAAAAAAAAAAAAAAAAAAAAAAAAAAAAAATGATTTTCAGTTC

>*Alu*Yb8_element_hg19_Chr11:90,444,512_population_analysis

ACAGGAAGGTTTAACTGATAGTGCCCTATCATATTAGTTCAGCAAAGAAAATCTATCTTTTGGCCGGGCGCGGTGGCTCACGCCTGTAATCCCAGCACTTTGGGAGGCCGAGGCGGGTGGATCATGAGGTCAGGAGATCGAGACCATCCTGGCTAACAAGGTGAAACCCCGTCTCTACTAAAAATACAAAAAATTAGCCGGGCGCGGTGGCGGGCGCCTGTAGTCCCAGCTACTCGGGAGGCTGAGGCAGGAGAATGGCGTGAACCCGGGAAGCGGAGCTTGCAGTGAGCCGAGATTGCGCCACTGCAGTCCGCAGTCCGGCCTGGGCGACAGAGCGAGACTCCGTCTCAAAAAAAAAAAAAAAAAAAAAAAAAAAAAAAAAAAAAAAAAAAAAAAAAAAAAAAAAAAAAAAAAAAAAAAAAAAAAAAGAAAATCTATCTTTTGAGAAAGTAGTGTTTCAATCCATATAAATAAATCTCATGTGGGTGAATTTCAGCATTATTTTAATAATAGGTATTATTTAAGACATTAATACATTCAAGACTTTGTGTAATGCAACAGGACACACAGATATATAGGGATGACTCTAATTTCAATAAGCTTATCCTCACAGGAAATAAATTTACATAAAGAATTCATATATGTATATGAAATCTTTAGAACTCTATCTAGCTATCTATATATATGATCATACGTAAATACATTATGTGAAAAATAT

>*Alu*Yb8_element_hg19_Chr12:1,762,791_doubleton_analysis

AATGAGTGAGGAGGGCAATGCAGTTAGGAAAGAAAACATGAAAGGAATGCCTTGTCCTGCTCGGCTGGACCCTCAAGACACCCACAGGCCGGGCGCGGTGGCTCACGCCTGTAATCCCAGCACTTTGGGAGGCCGAGGCGGGTGGATCATGAGGTCAGGAGATCGAGACCATCCTGGCTAACAAGGTGAAACCCCGTCTCTACTAAAAATACAAAAAATTAGCCGGGCGCGGTGGCGGGCGCCTGTAGTCCCAGCTACTCGGGAGGCTGAGGCAGGAGAATGGCGTGAACCTGGGAAGCGGAGCTTGCAGTGAGCCGAGATTGCGCCACTGCAGTCCGCAGTCCGGCCTGGGCGACAGAGCGAGACTCCGTCTCAAAAAAAAAAAAAAAAAAAAAAAAAAAAAAAAAAAAAAAGACACCCACAGTACTTTTCATGCTTGAAGATTATGATGAAGGAGGTGAGTAATAATTATAGCTACCATTTATCGAGTACTACTGTGTACCAGCTGCTTTACATACATTACATGTAATCCTCTCAGGGCCTCTGCAGTGTAGCCAATATTATCCACGTGTTTCAGATGGGAAAGCTAACACTCAGAGCGGTGAAGCGGCATCCCTCACCTCACACAGCTAGTAATTCATGCTGACTCCAGACCTCTTTCAGGTGGATTTCTGGGTAAGGAATCTGGTCAGCTGAAATAA

>*Alu*Yb8_element_hg19_Chr12:31,819,027_exon_analysis

GCCTGTTCCCAACATTTATTTTTCCTCATCTCTCTGAGACATGTTTCTCCTTTTTAATTTAGGTATCTTGGCCGGGCGCGGTGGCTCACGCCTGTAATCCCAGCACTTTGGGAGGCCGAGGCGGGTGGATCATGAGGTCAGGAGATCGAGACCATCCTGGCTAACAAGGTGAAACCCCGTCTCTACTAAAAAAAATACAAAAAATTAGCCGGGCGCGGTGGCGGGCACCTGTAGTCCCAGCTACTCGGGAGGCTGAGGCAGGAGAATGGCGTGAACCCGGGAAGCGGAGCTTGCAGTGAGCCGAGATTGCGCCACTGCAGTCCGCAGTCCGGCCTGGGCGACAGAGCGAGACTCCGTCTTAAAAAAAAAAAAAAAAAAAAAAAAAAAAAAAAAATTTAGGTATCTTTTGGATAATCCTGA

>*Alu*Yb8b1_element_hg19_Chr13:24,517,374_population_analysis

TATTTTCTAACTTCAGAAAGGCTTTTGTTTGAAAGGTGGGAGATAAAGTTTCAATGAGATTAAGTCCCGATATTCTCATTTTAAATCTCTCAGCTTGTGCAGGCAGGGAGGTAAACATGCAGTTTTTAAGGATGGAAGGGTTCTGAGAGAGAGAATACGTCTGCTACATAACAGGTACTCAGGTTATGTTTGATGAATAAATGGAATAAAAGAATAGATAAGGCCGGGCGCGGTGGCTCACGCCTGTAATCCCAGCACTTTGGGAGGCCGAGGCGGGTGGATCATGAGGTCAGGAGATCGAGACCATCCTGGCTAACAAGGTGAAACCCCGTCTCTACTAAAAATACAAAAAATTAGCCGGGCGCGGTGGCGGGCGCCTGTAGTCCCAGCTACTCGGGAGGCTGAGGCAGGAGAATGGCGTGAACCCGGGAAGCGGAGCTTGCAGTGAGCCGAGATTGCGCCACTGCAGTCCGCAGTCCGACCTGGGCGACAGAGCGAGACTCCGTCTCAAAAAAAAAAAAAAAAAAAAAAAAAAAAAAAAAAAAAGAATAGATAAATACAGTTGCGGAGTTCAGTATTTTTAAATAAACTCCTATAAAGCAATGTTTTTGCAATAGTAATTATTTGTGTTATTTTTATTTTTAAAGAATACAATTAAAATGAAATGATTAATCTATCATTGTTTGCATAAATGGAATGAATCTACACATAAGAAAAACACGTACGTAATAAATATATACATAATAACATCAGGAAACAGATAAAAACTTTCCATTTTCACTTAYGAAGAGGCTAAAAGTTCAAAGAAGATAATAACACACACAATAATGATAAAAAAAAGAAAGCGAGAAATTATTTTTTTCTGTGCAAGATTTATATTCTTCTCTTCCGAAAGATTATTCCAT

>*Alu*Yb8_element_hg19_Chr14:50,491,726_singleton_analysis

GGTCCAGCAGATCCCAGTTACCAGTGGGTAGAAGGATCTGGAAGGGTTGGGCAGGATGTGAAATAAGAAGCAATGATGGCCGGGCGCGGTGGCTCACGCCTGTAATCCCAGCACTTTGGGAGGCCGAGGCGGGTGGATCATGAGGTCAGGAGATCGAGACCATCCTGGCTAACAAGGTGAAACCCCGTCTCTACTAAAAATACAAAAAATTAGCCGGGCGCGGTGGCGGGTGCCTGTAGTCCCAGCTACTCGGGAGGCTGAGGCAGGAGAATGGCGTGAACCCGGGAAGTGGAGCTTGCAAGTGAGCCGAGATTGCGCCACTGCAGTCCGCAGTCCGGCCTGGGTGACAGAGCGAGACTCCGTCTCAAAAAAAAAAAAAAAAAAAAAAAAAAAAAAGAAGCAATGATGGTCTCAAGAACTGCTCAGGTGCTAGCAGTGCTAGGATGACG

>*Alu*Yb7.3_element_hg19_Chr14:63,572,168_replicate_analysis

CCACAATCTATGCCCTGAAATATTATGCTATCTCAACACATGTTTTTAGAATTAACTTTAGGCCGGGCGCGGTGGCTCACGCCTGTAATCCCAGCACTTTGGGAGGCCGAGGCGGGTGGATCACGAGGTCAGGAGATCGAGACCATCCTGGCTAACAAGGTGAAACCCCGTCTCTACTAAAAATACAAAAAATCAGCCGGGCGCGGTGGCAGGCGCCTGTAGTCCCAGCTACTCGGGAGGCTGAGGCAGGAGAATGGCGTGAACCCGGGAAGCAGAGCTTGCAGTGAGCCGAGATTGCGCCACTGCAGTCCGCAGTCCGGCCTGGGCGACAGAGCGAGACTCCGTCTCAAAAAAAAAAAAAAAAAAAAAAAAAAAGAATTAACTTTAGTTAATTTAAAGTCTTTATTTAAGTCTTTATTTAAATAATTTCAAG

>*Alu*Yb8_element_hg19_Chr14:64,520,979_singleton_analysis

GGAGTGTGTTGGCGTCACTTTCCAACAGAGGTGAGGAAATAATCACAACTTACTCAACTCTGCTGCTATAGCATGAAAACAGATACAGTCAATACAAAAACAAATGGGTATGGCTATATTCCAATAAAACTTTATTTATAAAAAACAGGCACTGCGGGAAGGATTTGGCCCATGGACCATGAGCATGTGATGCTCCCTGCTCTGGTTCTGTTACTGTTCTCAGGACTCTGAGAAAAATCTTCATGTTTCTAAACTTCAATTTCCTTATTTTTAAACGGGGCTAATAATAGCACTCATCTCACAGAGTTGCTATAAAAATTCAACATGAAATTTTTAGCACAGTGTCTCCAACACTGTAAGTTCTACATAAATGTTAGTTATTATTTGGTGTTCTTCCCTCTACACCATAATTCTTCCATTATATCTTACTACCTCTAAAAACAAAACAGAGGCCGGGCGCGGTGGCTCACGCCTGTAATCCCAGCACTTTGGGAGGCCGAGGCGGGTGGATCATGAGGTCAGGAGATCGAGACCATCCTGGCTAACAAGGTGAAACCCCGTCTCTACTAAAAATACAAAAAATTAGCCGGGCGCGGTGGCGGGCGCCTGTAGTCCCAGCTACTCGGGAGGCTGAGGCAGGAGAATGGCGTGAACCCGGGAAGCGGAGCTTGCAGTGAGCCGAGATTGCGCCACTGCAGTCCGCAGTCCGGCCTGGGCGACAGAGCGAGACTCCGTCTCAAAAAAAAAAAAAAAAAAAAAAAAAAAAAAAAAAAAAAAAAAAAAAAAAAAAAAAAAAAAAAAAAAAACAAAACAGAAAACAAATTTTTAAAAATGTTCTGAGCTCAACAGAGACTACGAGGAAAACAGAAATAAGAGTCAGA

>*Alu*Yb8_element_hg19_Chr14:76,353,218_singleton_analysis

>Appears to be clonal populations with A or G in 92-40-35

CTGAGGACGACAGCCTACAAACAACTACATGCATCTGAACTGTCTCTTGTAAATGAGCTTTTTTCAGAGCCAGAATCATACTCTCCAGGAAATATGGAGAAAGAAACCTGAGGAGGCTGGGCGCGGTGGCTCACGCCTGTAATCCCAGCACTTTGGGAGGCCGAGGCGGGTGGATCATGAGGTCAGGAGATCGAGACCATCCTGGCTAACAAGGTGAAACCCCGTCTCTACTAAAAATACAAAAAAAATTAGCCGGGCGCGGTGGCGGGCGCCTGTAGTCCCAGCTACTCGGGAGGCTGAGGCAGGAGAATGGCGTGAACCCGGGAAGCGGAGCTTGCAGTGAGCCGAGATTGCRCCACTGCAGTCCGCAGTCCGGCCTGGGCGACAGAGCGAGACTCCGTCTCAAAAAAAAAAAAAAAAAAAAAAAAAAAAAAAAAAAAAGAAAGAAACCTGAGGAGATTGAAGTTTGCCAGGCACAAGGGCAAAACTCAGACTGAATGAATTTGAAAGGGTGGGGCCAAAGATGTTGTAACCTGGGAGACTTCTCTGAAGAAAGAAAACTGTTTAAGAAACACAGACTGAACTGCATACTTTTCCTTAAATAGCTGAGATGACCTTCTTTACCCTGGGCTTAGGTGA

>*Alu*Yb8b1_element_hg19_Chr15:56,762,806_replicate_analysis

GATGCTGCCTTTATGCCTGCATCAGTTTTCCCTAGCATTCCCCATACTTCCAAATTAGGAAGTCTCTGCTTCTAACAAAATATCGTTGTTATTGCTGATATGGACACAAATCAGCAAGTACTGGATACCTAATACAATTTAGTAATTGTCATTATTGTTCTATAAATACTATGTAGAACATATATTGTATAATATTCTATGAGCACATCGCATTTCATTATGTATAAATTGTTATATCATCTATGATACTGTTAACATGATATCTTTTATTACAGTCAGAGTAATTAATTGTAATACAAATTTTAATAAAACAAAGCTGGGTTTGCCTAGGAAAGAGTAAATGAGGCCGGGCGCGGTGGCTCACGCCTGTAATCCCAGCACTTTGGGAGGCCGAGGCGGGTGGATCATGAGGTCAGGAGATCGAGACCATCCTGGCTAACAAGGTGAAACCCCGTCTCTACTAAAAATACAAAAAATTAGCCGGGCGCGGTGGCGGGCGCCTGTAGTCCCAGCTACTCGGGAGGCTGAGGCAGGAGAATGGCGTGAACCCGGGAAGCGGAGCTTGCAGTGAGCCGAGATTGCGCCAACTGCAGTCCGCAGTCCGACCTGGGCGACAGAGCGAGACTCCGTCTCAAAAAAAAAAAAAAAAAAAAAAAAAAAAAAAAAAAAAAAAAAAAAGAAAGAGTAAATGAGGAATCACTTTGGTTTACTACTCTATAATTATTGGAAGGATTGGTACAATTAGTTGAAAATACTTCAAATATGTCTGATTCAG

>*Alu*Yb8_element_hg19_Chr18:55,575,980_replicate_analysis

TTCGAGAAAGAAAGGTCCGGAAAAGCATCCCTAAAGACTCAAATATTCCAAGGAGCAAAAAATATAGATAAGACAAAGCTCCAAAGACTCCTAACAGTGGAAATAGCTGTCAGATGTTATAGAGAAGGGAGCTGATGGGTTCCCACATGCTATTAACAAACCATGAAGAAGTCCCGGCCGGGCGCGGTGGCTCACGCCTGTAATCCCAGCACTTTGGGAGGCCGAGGCGGGTGGATCATGAGGTCAGGAGATCGAGACCATCCTGGCTAACAAGGTGAAACCCCGTCTCTACTAAAAATACAAAAAATTAGCCGGGCGCGGTGGCGGGCGCCTGTAGTCCCAGCTACTCGGGAGGCTGAGGCAGGAGAATGGCGTGAACCCGGGAAGCGGAGCTTGCAGTGAGCCGAGATTGCGCCACTGCAGTCCGCAGTCCGGCCTGGGCGACAGAGCGAGACTCCGTCTCAAAAAAAAAAAAAAAAAAAAAAAAAAAAAAAAAAAAAAGAAGTCCCTTAGCCTTTGGACCTGGAGT

>*Alu*Yb5_element_hg19_Chr19:53,600,715_doubleton_analysis

TCCACAGCGGGTTTCAAGGACCTTGCCCCTCAGCTCCCCAGCTGCCTTGTGACTCTGTGACTCTGTTATCTTGGAATAAACAGGGAGAGGCCACCGGCCTGGCTGCAAGAATGTGGCAGCTTTATCTTCCCAAGGACAGTCCCAGTGGAAACCTCTGGGCCGCAGGTCAGTGTGCTGTGTTTCACTCTGATTCTTGGAGAAATCTTTCCCTTACTCTGGGACCCTGATAATCACTCCCTCTTGAGTTGCACCCACAGACGCCCCCACAACAAACCCTCCCTAATGAAAGCCATCTGCGCCTGATTCTTAGGATCCCCGTTGCCCTCTGGACCCAGACACGCTTACAAACTCCCACACTCCCCATATACTTCATAAAGATCCCACAATCGGCCGGGCGCGGTGGCTCACGCCTGTAATCCCAGCACTTTGGGAGGCCGAGGCGGGTGGATCACGAGGTCAGGAGATCGAGACCATCCTGGCTAACGTGGTGAAACCCCGTCTCTACTAAAAATACAAAAAATTAGCCGGGCATGGTGGCGGGCGCCTGTAGTCCCAGCTACTCGGGAGGCTGAGGCAGGAGAATGGCGTGAACCCGGGAGGCGGAGCTTGCAGTGAGCTGAGATTGCGCCACTGCAGTCCGCAGTCCGGCCTGGGCGACAGAGCGAGACTCCGTCTCAAAAAAAAAAAAAAAAAAAAAAAAAAAAAAAAAAGATCCCACAATCCCCAGATACTTCATAAAGATCCAGAAGCCCTGTTATGAAATCTCACAAGCCCCAGTAACTCATGGAATAATATACACAAACACCCCAGGAACTCACTGACCCAGGGCAACGTATTTCCACTCATAGTGACTCCACCCCACAGGGGAATACCACTGAAACCTACCAATAAAACAGAGA

>*Alu*Yb8b1_element_hg19_Chr20:55,427,799_singleton_analysis

GCAGCTACGGAGGGAAAAGGGCCGTCTTGATCCTCACTGTTTTGAATGTGTAAAGTGGGGGTAAGGTGGAGGTAGGGGGCTCTCTGCAGAGCGACACCCAGGCTGGGCACTGGTCACTGTGTTGTGGGCCTTACATTCTTTCGCTAGGCCTCCAAACCACCGTTAAGAGGTAGGAAGGCCGGGCGCGGTGGCTCACGCCTGTAATCCCAGCACTTTGGGAGGCCGAGGCGGGTGGATCATGAGGTCAGGAGATCGAGACCATCCTGGCTAACAAGGTGAAACCCCGTCTCTACTAAAAATACAAAAAATTAGCCGGGCGCGGTGGCGGGCGCCTGTAGTCCCAGCTACTCGGGAGGCTGAGGCAGGAGAATGGCGTGAACCCGGGAAGCGGAGCTTGCAGTGAGCCGAGATTGCGCCACTGCAGTCCGCAGTCCGACCTGGGCGACAGAGCGAGACTCCGTCTCAAAAAAAAAAAAAAAAAAAAAAAAAAAAAAAAAAAAGAGGTAGGAACTATCTTTAAGTCCATTTCACAGACTAACCACTAAGGCACAATGAAGTAACCTGCCCAAGGTCAAGTATCTGGTAACTACAA

>*Alu*Yb7.4_element_hg19_ChrX:9,116,091_population_analysis

ATCCACTGACAGAAGCCGGCTTTGTTTTTAAAAACTTTTCCTGGCCGGGCGCGGTGGCTCACGCCTGTAGTCCCAGCACTTTGGGAGGCCGAGGCGGGTGGATCATGAGGTCAGGAGATCGAGACCATCCTGGCTAACAAGGTGAAACCCCGTCTCTACTAAAAAATACAAAAAATTAGCCGGGCGCGGTGGCGGGCGCCTGTAGTCCCAGCTACTCGGGAGGCTGAGGCAGGAGAATGGCTTGAACCCGGGAGGCGGAGCTTGCAGTGAGCCGAGATTCCGCCACTGCAGTCCGCAGTCCGGCCTGGGCGACAGAGCGAGACTCCGTCTCAAAAAAAAAAAAAAAAAAAAAAAAAAAAACTTTTCCTGTAATAGCTCACCGAGGTCAGGCCTCAGCTCTTGTAGGCAGCTCTCATAAACT

>*Alu*Yb8_element_hg19_ChrX:94,665,935_population_analysis

TAGCAGCACCCCTGCTCTACTGGTACCAATTTACCATATTAGTCCATTTTCATGCTGCTGATAGTGACATATCTGAGACTGGGTAATTTATAAAGAAAAAGAGGGCCGGGCGCGGTGGCTCACGCCTGTAATCCCAGCACTTTGGGAGGCCGAGGCGGGTGGATCATGAGGTCAGGAGATCGAGACCATCCTGGCTAACAAGGTGAAACCCCGTCTCTACTAAAAATACAAAAAATTAGCCGGGCGCGGTGGCGGGCGCCTGTAGTCCCAGCTACTCGGGAGGCTGAGGCAGGAGAATGGCGTGAACCCGGGAAGCGGAGCTTGCAGTGAGCTGAGATTGCGCCACTGCAGTCCGCAGTCCGGCCTGGGCGACAGAGCGAGACTCCGTCTCAAAAAAAAAAAAAAAAAAAAAAAAAAAAAAAAAAAAAAAAAAAAAAAAAAAAAAAAAAAAAAAAAAAAAAGAAAAAGAGGTT

>*Alu*Yb8_element_hg19_ChrY:9,992,131_population_analysis

TGAAGAAGGTGGGGAGGAGTAGAAGAAAAAATATTTGAAAAAACTGTGGCTGAGGCCGGGCGCGGTGGCTCACGCCTGTAATCCCAGCACTTTGGGAGGCCGAGGCGGGTGGATCATGAGGTCAGGAGATCGAGACCATCCTGGCTAACAAGGTGAAACCCCGTCTCTACTAAAAATACAAAAAATTAGCCGGGCGCGGTGGCGGGCGCCTGTAGTCCCAGCTACTCGGGAGGCTGAGGCAGGAGAATGGCGTGAACCCGGGAAGCGGAGCTTGCAGTGAGCCGAGATTGCGCCACTGCAGTCCGCAGTCCGGCCTGGGCGACAGAGCGAGACTCCGTCTCAAAAAAAAAAAAAAAAAAAAAAAAAAAAAAAAAAACTGTGGCTGAAAAAATT
